# Re-evaluating Scythian lifeways: Isotopic analysis of diet and mobility in Iron Age Ukraine

**DOI:** 10.1371/journal.pone.0245996

**Published:** 2021-03-10

**Authors:** Alicia R. Ventresca Miller, James Johnson, Sergey Makhortykh, Claudia Gerling, Ludmilla Litvinova, Svetlana Andrukh, Gennady Toschev, Jana Zech, Petrus le Roux, Cheryl Makarewicz, Nicole Boivin, Patrick Roberts

**Affiliations:** 1 Department of Anthropology and Museum of Anthropological Archaeology, University of Michigan, Ann Arbor, Michigan, United States of America; 2 Max Planck Institute for the Science of Human History, Department of Archaeology, Stable Isotope Group, Jena, Germany; 3 Graduate School of Human Development in Landscapes, Kiel University, Kiel, Germany; 4 Institute for Prehistoric and Protohistoric Archaeology, Kiel University, Kiel, Germany; 5 Department of Anthropology, University of Wyoming, Laramie, Wyoming, United States of America; 6 Institute of Archaeology of National Academy of Sciences Ukraine (NUAS), Kyiv, Ukraine; 7 Department of Environmental Sciences, Integrative Prehistory and Archaeological Science, University of Basel, Basel, Switzerland; 8 Zaporizhzhya National University, Zaporizhzhya, Ukraine; 9 Department of Geological Sciences, University of Cape Town, Rondebosch, South Africa; 10 School of Social Science, University of Queensland, Brisbane, Australia; 11 Department of Archaeology, University of Calgary, Calgary, Canada; 12 Smithsonian Institution, New York, NY, United States of America; Museo delle Civiltà, ITALY

## Abstract

The Scythians are frequently presented, in popular and academic thought alike, as highly mobile warrior nomads who posed a great economic risk to growing Mediterranean empires from the Iron Age into the Classical period. Archaeological studies provide evidence of first millennium BCE urbanism in the steppe while historical texts reference steppe agriculture, challenging traditional characterizations of Scythians as nomads. However, there have been few direct studies of the diet and mobility of populations living in the Pontic steppe and forest-steppe during the Scythian era. Here, we analyse strontium, oxygen, and carbon isotope data from human tooth enamel samples, as well as nitrogen and carbon isotope data of bone collagen, at several Iron Age sites across Ukraine commonly associated with ‘Scythian’ era communities. Our multi-isotopic approach demonstrates generally low levels of human mobility in the vicinity of urban locales, where populations engaged in agro-pastoralism focused primarily on millet agriculture. Some individuals show evidence for long-distance mobility, likely associated with significant inter-regional connections. We argue that this pattern supports economic diversity of urban locales and complex trading networks, rather than a homogeneous nomadic population.

## Introduction

Public and academic perceptions of the Scythian-era Eurasian steppe (ca. 700 to 200 cal BCE), from the northern Black Sea to the Altai Mountains, have frequently highlighted its domination by warrior nomads [1–4]. This stereotype is documented as far back as Herodotus (Chapter IV, *Histories*), who describes Scythian populations living in wagons while also engaging in raiding and warfare [5,6]. Archaeologically, this view has been supported by material evidence for horse harnesses, weapons, monumental burial mounds, and a shared set of ‘animal style’ ornaments and belt plaques [3] identified across the Eurasian steppe. Yet, the adoption of this warrior nomad narrative by academics has resulted in diverse cultures and periods being lumped together as a homogeneous Scythian ‘culture’ [3,7] or, even more controversially, as parts of a vast Scythian ‘Empire’ encompassing the whole of the Eurasian steppe [2]. In reality, there are clear indications of variability in the historical designations (e.g. Scythian, Sarmatian, and Saka) used for populations who engaged in horseback riding, had armaments, and are purported to have roamed the steppe in different periods of time and locales.

**Fig 1:**
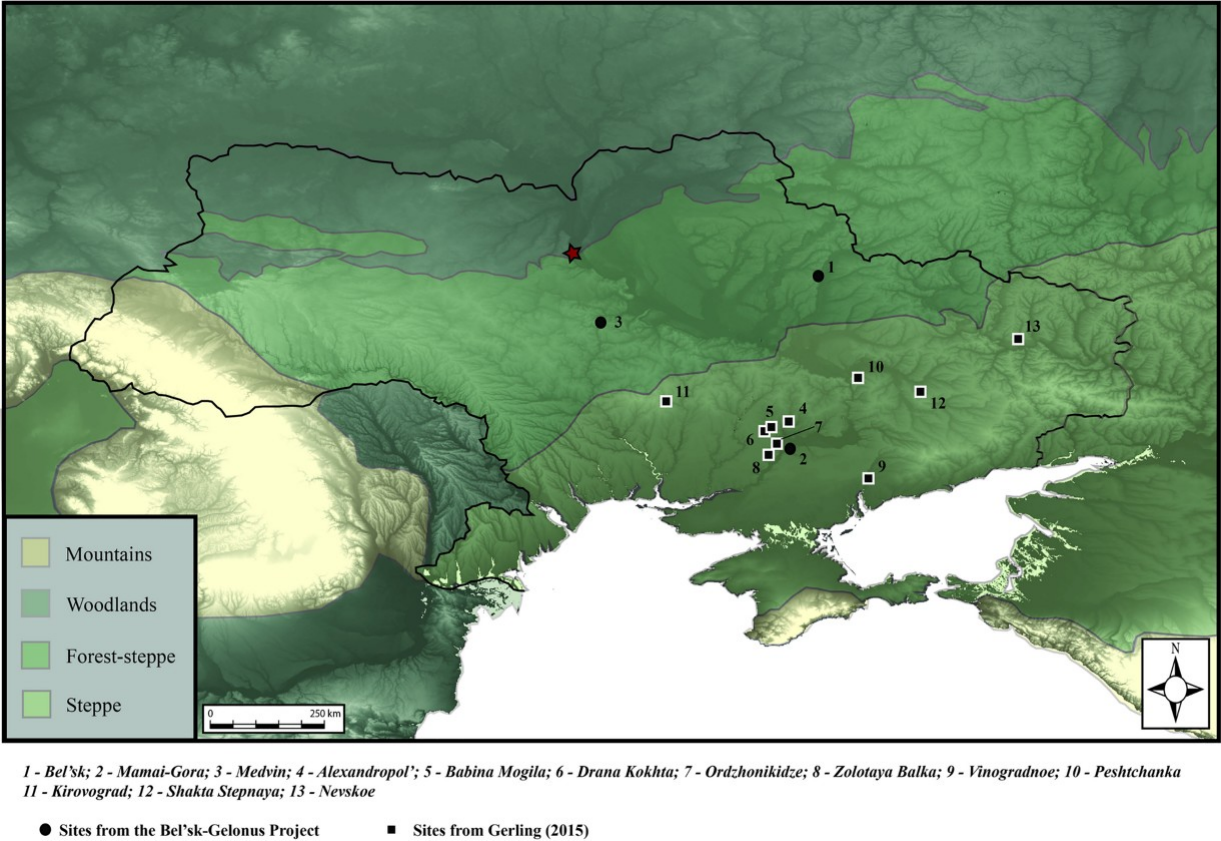
Environmental zones of Ukraine and surrounding regions identifying zones of woodland, forest-steppe, steppe, and mountainous zones (imposed over SRTM DEM mosaic) with sites in text identified. Red star is the location of Kyiv. Basemap constructed in ArcGIS 10 as mosaic using data downloaded from CGIAR Consortium for Spatial Information (https://cgiarcsi.community). Site locations and ecological zones generated in Adobe Illustrator CC 2020.

Descriptions of mobile populations and warrior nomads have overshadowed archaeological and textual evidence illustrating that the Pontic region was inhabited by farmers and pastoralists, as well as Scythian warriors and ‘Royal’ Scythians [2,5,6,8–12]. Moreover, archaeological evidence lends support for diverse subsistence and productive (iron, ceramic) economies, which some have linked to environmental variation, with agro-pastoralists occupying the forest-steppe and nomads the steppe proper [6,12]. The Scythian era site of Bel’sk, a large complex of cemeteries and settlements, is larger than most urban centers in Iron Age Europe [13] and contemporaneous with other urban locales in the Pontic steppe (Fig 1). This attests to the array of mobile pastoralists living alongside sedentary, presumably agricultural or agro-pastoral populations, rather than a region of roaming hordes. Research on the subsistence economies of populations in the Pontic steppe indicates that pastoralism was an important part of the economy. However, archaeobotanical evidence of domesticated crops has been recovered from Iron Age contexts [14–18], with millet, wheat, and barley identified at sites across Ukraine as early as the Neolithic [19,20].

The persistence of the idea of ‘nomadic’ Scythians in popular and academic thought, in the face of these growing datasets, lies in the fact that open steppe-lands are frequently seen as key crossroads for population movement, with the spread of animal style motifs lending support to narratives of mobile nomad warriors engaging in long distance east-west interactions [3]. Yet, recent genetic studies suggest that extensive human migration was in fact higher in periods *predating* the Iron Age, and actually decreased during the Iron Age itself [21–23]. Genetic evidence from Iron Age populations across the steppe indicates a clear demographic separation between Hungarian and Inner Asian populations in the first millennium BCE [24]. Furthermore, Scythian era populations from south central Ukraine fall into three main groups of ancestry (referred to as west Eurasian hunter-gathers, Neolithic Anatolian farmers, and East Eurasian lineages), with evidence for substantial gene flow into the region before the dawn of the Iron Age [21–23]. Here, we seek to add to emerging insights into Scythian lifeways through a multi-isotopic approach which provides direct evidence for mobility and dietary intake.

## Materials and methods

### Cultural background

The Eneolithic period in the North Pontic Region lasts from 3900 to 2900 cal BCE [25] and includes the well-known Tripolye culture. This is a period often discussed as one of intense migration and nomadism, while the economy is considered to have been based on pastoralism. The Early Bronze Age Yamnaya period spans from 3300 to 2500 cal BCE and is distributed over a vast territory, with regional variation in material culture [26]. The subsistence economy was based primarily on pastoralism–with some scholars suggesting these populations were highly mobile or semi-nomadic [27]–alongside some initial cultivation of wheat, barley, hemp, millet [28]. The Early Catacomb and Catacomb cultures spanned 2700 to 2200 cal BCE and 2200 to 2000 cal BCE, respectively [29]. The economy of these groups was mixed, while primarily pastoral they also practiced agriculture [28]. Finally, the Iron Age period ranges from 700 to 200 cal BCE and is characterized by the Scythian culture groups. The economy of these groups has previously been linked to nomadic pastoralism, with high levels of mobility, however some scholars suggest that large settlements are evidence of a sedentary component of society that engaged in agricultural activities [30].

The sites analysed in this study date to the Iron Age (700–200 cal BCE), an era characterized by Scythian material culture. Relative dating at Medvin suggests that the burials are from two chronological periods, from the 7^th^ to 6^th^ or 5^th^ to 4^th^ centuries BCE [31–33], recently confirmed by our absolute dates (775–431 and 484–233 cal BCE) as well as published dates (756–413, 788–537 and 758–416 cal BCE) [34]. The site has three mound groups, with the materials analysed belonging to group 1 which is made up of 25 small mounds (height) and covers an area 135 x 70 meters. Mounds, or kurgans, vary in size with the largest reaching 1.7 m in height and 23 m in diameter [32]. The graves are described as neither elite nor commoner, but having an intermediate status [32,33]. There was no settlement in close proximity to the cemetery.

Bel’sk is a massive fortified complex that includes multiple settlements and cemeteries. The fortifications are dated to the 6^th^ century BCE and link three fortified settlements [35–37]. The site of Bel’sk overlaps chronologically with Medvin, dating from c. 700 to 400 BC [38–41], with radiocarbon results confirming this general range of dates (782–480 and 794–536 cal BCE) (S1 Text in S1 File). Over a thousand burial mounds (kurgans) have been identified through archaeological survey, geophysics, and historical documentation [39,40,42–44]. Several cemeteries are located both within the fortification (Cemetery A and Cemetery B) and just outside the enclosure (Pereschepino, Osnyagi, Skorbor, and Marchenki), with additional skeletal remains recovered from the Tsarina settlement. We analyzed Scythian era individuals from Pereschepino, Cemetery B, Tsarina, Osnyagi, and Marchenki.

The site of Mamai-Gora includes burials from the Neolithic through Golden horde era. The Iron Age component of the site dates from the end of the 5th through the 2^rd^ century BCE, based on detailed analyses of material culture from burials [45–49] and our recent radiocarbon dates (399–209 and 369–174 cal BCE) (S1 Text in S1 File). In addition, recently published dates from the site indicate that burials date similarly or slightly earlier (366–171 and 725–394 cal BCE) [34]. Over 320 burials have been identified at the site of Mamai-Gora making it one of the largest cemeteries in the region. Kurgan (mounds) at the site were impacted by agricultural activities and the construction of water containment areas and reservoirs in the modern era [46]. Kurgans included multiple burials and graves held from one to several individuals, ranging in size from five to over 50 m in diameter [45–48].

### Radiocarbon dating

We dated four samples from the sites of Mamai-Gora (n = 2) and Medvin (n = 2). Samples were AMS ^14^C dated following standard protocol at the Poznan Radiocarbon Laboratory. See Supplemental Text (S1 Text in S1 File)

### Bioarchaeological methods

Before sampling, we evaluated the biological sex of sampled adult humans using morphological features and metrical criteria specific to the pelvis and cranium [50–52]. Age-at-death was estimated using multiple methods as per [52]. Among adults, age-at-death was assessed by auricular surface and pubic symphysis degeneration. For subadults, methods included timing of tooth eruption, age at epiphyseal fusion, and evidence from primary ossification centers (Tables 1, 2, S1a-S1e and S3 Tables in S1 File).

**Table 1:** Isotope values for individuals from the site of Medvin.

Sample Number	Individual Number	Cemetery	Grave	Individual	# of paired teeth	Sex	Age Range	^87^/^86^Sr (M1)	^87^/^86^Sr (M2)	^87^/^86^Sr (M3)	Sr difference between paired teeth	δ^18^O_(C-VDPB)_ (M1)	δ^18^O_(C-VDPB)_ (M2)	δ^18^O_(C-VDPB)_ (M3)	δ^18^O difference between paired teeth	δ^13^C_(C-VDPB)_ (M1)	δ^13^C_(C-VDPB)_ (M2)	δ^13^C_(C-VDPB)_ (M3)	δ^13^C difference between paired teeth	δ^15^N (collagen)	δ^13^C (collagen)
223	23	Medvin	K 8, Grave 1	1 of 1	−	Female	> 40 yrs		−	0.7108	−	−	−	−	−	−	−	−	−	10.6	-13.9
225, 226	24	Medvin	K-13, grave 1	1 of 1	2	Male	> 50 yrs	0.7107	−	0.7109	0.0002	−	−	−	−	−	−	−	−	−	−
228, 229	25	Medvin	K 6, Grave 1	1 of 1	2	Male	40–50 yrs	0.7106	−	0.7106	0.0001	-5.7	−	-6.4	0.8	-6.7	−	-4.1	2.6	10.8	-13.1
231, 232	26	Medvin	K 9, Grave 1	1 of 3	2	Male	24–35 yrs	0.7110	−	0.7108	0.0001	-5.7	−	-6.7	1.0	-7.3	−	-9.1	1.8	9.7	-14.9
234, 235	27	Medvin	K 9, Grave 1	2 of 3	2	Indet.	35–40 yrs	0.7111	−	0.7108	0.0003	-5.2	−	-5.8	0.6	-7.0	−	-9.8	2.8	10.3	-16.8
237, 238	28	Medvin	K 9, Grave 1,	3 of 3	2	Indet.	30–35 yrs	0.7106	−	0.7107	0.0000	-6.3	−	-6.6	0.3	-8.0	−	-6.8	1.2	9.6	-15.7
240, 241	29	Medvin	K 1, Grave 1	1 of 1	2	Male	> 30 yrs	0.7108	0.7106	−	0.0002	-4.9	-7.4	−	2.6	-7.1	-3.4	−	3.8	11.0	-13.7
242	−	Medvin	K 22, Grave 1	2 of 6	−	Male	> 45 yrs	−	−	−	−	−	−	−	−	−	−	−	−	10.9	-15.6
243	−	Medvin	K 22, Grave 1	1 of 6	−	Male	> 55 yrs	−	−	−	−	−	−	−	−	−	−	−	−	10.1	-14.6
246	30	Medvin	K 22, Grave 1	4 of 6	−	Subadult	6–10 yrs	0.7108	−	−	−	-5.3	−	−	−	-8.7	−	−	−	10.0	-15.3
248	31	Medvin	K 22, Grave 1	3 of 6	−	Subadult	4–8 yrs	0.7108	−	−	−	-6.0	−	−	−	-6.7	−	−	−	10.4	-15.0
**Average**	** **	** **	** **	** **	** **	** **	** **	0.7108	0.7106	0.7108	**0.0002**	-5.6	-7.4	-6.4	**1.0**	-7.4	-3.4	-7.4	**2.4**	10.3	-14.9

**Table 2:** Isotope values for individuals from the site of Mamai-Gora.

Sample Number	Individual Number	Cemetery	Grave	Individual	# of paired teeth	Sex	Age Range	^87^/^86^Sr (M1)	^87^/^86^Sr (M2)	^87^/^86^Sr (M3)	Sr difference between paired teeth	δ^18^O_(C-VDPB)_ (M1)	δ^18^O_(C-VDPB)_ (M2)	δ^18^O_(C-VDPB)_ (M3)	δ^18^O difference between paired teeth	δ^13^C_(C-VDPB)_ (M1)	δ^13^C_(C-VDPB)_ (M2)	δ^13^C_(C-VDPB)_ (M3)	δ^13^C difference between paired teeth	δ^15^N (collagen)	δ^13^C (collagen)
174, 176	32	Mamai-Gora	K. 110, Grave 2	1 of 1	2	Male	17–40 yrs	0.7099	−	0.7100	0.0000	-5.0	−	-7.2	2.2	-11.7	−	-11.0	0.7	12.5	-16.3
183	−	Mamai-Gora	K. 125	1 of 1	−	Male	28–55 yrs	−	−	−	−	−	−	−	−	−	−	−	−	12.5	-16.3
185, 186	33	Mamai-Gora	K. 114, Grave 3	1 of 1	2	Male	20–35 yrs	0.7098	−	0.7099	0.0001	-5.9	−	-6.6	0.6	-12.0	−	-7.4	4.6	11.2	-15.5
188, 189	34	Mamai-Gora	K. 106, Grave 1	1 of 1	2	Male	40–50 yrs	0.7098	0.7098	−	0.0001	-7.1	-5.0	−	2.0	-8.1	-10.7	−	2.6	−	−
191, 192	35	Mamai-Gora	K. 109, Grave 2	1 of 1	2	Female	30–45 yrs	0.7102	0.7099	−	0.0004	-3.9	-5.7	−	1.8	-11.0	-6.7	−	4.3	12.7	-16.5
194, 195	36	Mamai-Gora	K. 103, Grave 1	1 of 1	2	Female	24–30 yrs	0.7099	−	0.7099	0.0000	-5.0	−	-6.1	1.2	-9.0	−	-9.5	0.5	12.0	-17.7
197, 198	37	Mamai-Gora	K. 160, Grave 1	1 of 2	2	Subadult	3.5–6.5 yrs	0.7100	−	−	−	-5.6	-5.3	−	0.3	-8.6	-10.2	−	1.6	14.6	-14.9
200, 201	38	Mamai-Gora	K. 160, Grave 1	2 of 2	2	Male	21–35 yrs	0.7091	−	0.7104	0.0013	-5.4	−	-6.2	0.8	-10.1	−	-11.2	1.1	12.3	-17.4
203, 204	39	Mamai-Gora	K. 160, Grave 2	1 of 1	2	Female	15–23 yrs	0.7098	−	0.7099	0.0001	-5.8	−	-6.8	1.1	-7.5	−	-8.7	1.2	11.5	-16.0
206, 207	40	Mamai-Gora	K. 63, Grave 1	1 of 1	2	Male	>35 yrs	0.7100	0.7100	−	0.0000	-4.4	-5.8	−	1.4	-7.3	-11.1	−	3.8	12.9	-15.8
209, 210	41	Mamai-Gora	K. 20, Grave 2	1 of 1	2	Male	30–40 yrs	0.7099	−	0.7101	0.0002	-5.4	−	-6.8	1.3	-9.2	−	-10.9	1.8	11.1	-16.8
211	−	Mamai-Gora	K. 132, Grave 1	1 of 1	−	Male	30–40 yrs	−	−	−	−	−	−	−	−	−	−	−	−	11.7	-17.4
213, 214	42	Mamai-Gora	K. 128, Grave 1	1 of 1	2	Male	20–40 yrs	0.7099	−	0.7101	0.0002	-6.8	−	-5.6	1.2	-12.2	−	-11.6	0.5	11.7	-17.5
215	−	Mamai-Gora	K. 153, Grave 1	1 of 1	−	Male	40–50 yrs	−	−	−	−	−	−	−	−	−	−	−	−	12.7	-16.8
216, 217	43	Mamai-Gora	K. 82, Grave 2	1 of 1	2	Female	35–45 yrs	0.7111	0.7113	−	0.0002	-6.1	-6.0	−	0.1	-12.3	-10.9	−	1.4	11.5	-17.5
220, 221	44	Mamai-Gora	K. 58, Grave 2	1 of 1	2	Male	40–50 yrs	0.7100	−	0.7099	0.0000	-4.6	−	-5.7	1.0	-9.8	−	-11.1	1.3	12.6	-16.7
**Average**	** **	** **	** **	** **	** **	** **	** **	0.7100	0.7102	0.7100	**0.0002**	-5.5	-5.6	-6.4	**1.2**	-9.9	-9.9	-10.2	**2.0**	12.2	-16.6

Human remains are in a repository at the Institute of Archaeology of the Ukrainian Academy of Sciences in Kyiv. With permission from the Institute of Archaeology, we sampled dentition (teeth) and bone from the sites of Mamai-Gora, Medvin and Bel’sk. Our sampling was permitted through a signed ‘Agreement Concerning Scientific Collaboration’. When possible, two permanent molars were sampled from each individual, with a preference for the mandibular M1 and M3, then the maxillary M1 and M3. In cases when the M3 was absent, we collected the M2, and in some cases only a single molar was available. As our project was concerned with strontium isotope variation, we selected the M1 and M3 to explore differences over the life of the individual. The first molar (M1) crown mineralizes by 2.5 to 3 years of age, the crown of the second molar (M2) mineralizes by 7 to 8 years of age, and the third molar (M3) crown mineralizes by 12 to 16 years of age [53]. While tooth selection was preferred from the left mandible, this was not standard across individuals due to issues with skeletal preservation.

Samples of human bone were also collected from each individual with a preference for rib bones and secondarily from long bones. Bone from faunal material recovered from burial context was scarce, but included 3 samples of sheep (n = 2) and a pig.

### Sampling for isotope analysis

**Fig 2:**
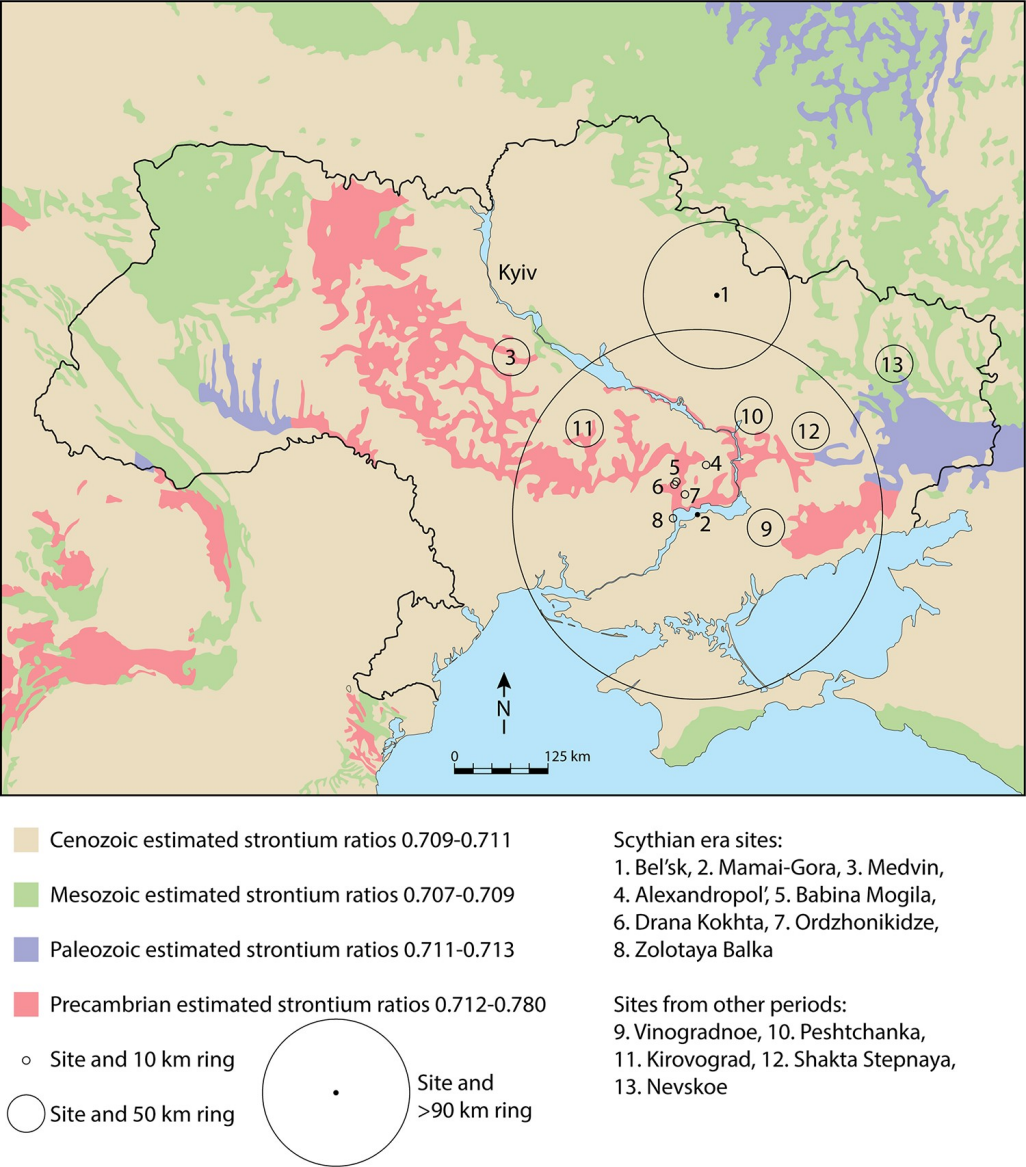
Map of geologic substrates in Ukraine and estimation of the extent of human mobility (circles) at each site. Basemap constructed in Adobe Illustrator CC 2020 from available geologic maps.

We performed isotopic analysis on enamel (^87^Sr/^86^Sr, δ^13^C, δ^18^O) and bone (δ^13^C, δ^15^N) from human skeletons (n = 56) and fauna (n = 3) recovered from archaeological sites located in the Pontic forest-steppe (Medvin, Bel’sk) and steppe (Mamai-Gora). At the site of Medvin, we sampled 11 individuals (specimens of 15 teeth/10 bone), at Mamai-Gora we sampled 16 individuals (specimens of 25 teeth/15 bone) and at Bel’sk we sampled 29 individuals and 3 fauna (specimens of 32 bone). We compared this to previously published data compiled from Neolithic through Iron Age populations [54–56] across Ukraine, in order to directly test the degree of mobility and dietary reliance of Scythian era populations. Human isotope data provide insights into mobility and subsistence at the level of the individual. Strontium isotope analysis provides direct evidence for human mobility, as ^87^Sr/^86^Sr ratios in food and drink varies broadly in accordance with geology (Fig 2), with distinctions being passed on to consumers [54,57–64].

Stable oxygen isotope measurements of human enamel offer further insight into human mobility and landscape use [65–67] as they primarily reflect imbibed water [68–71] with some caveats (see methods). Stable carbon isotope analysis of enamel bioapatite has been used to reconstruct ancient human diets revealing the extent of reliance on domesticated C_4_ crops such as millet or maize, the latter being more relevant to the Americas [72–75]. Stable carbon and nitrogen analysis of bone collagen has been used to reconstruct dietary intake of humans, especially in comparison with fauna from the same locale and period [76]. Bone collagen δ^13^C primarily tracks the protein portion of the diet, while tooth enamel bioapatite δ^13^C reflects the whole diet [77].

### Strontium isotope ratios of ancient humans

We sampled a total of 9 human individuals (15 teeth) from Medvin and 13 human individuals (25 teeth) from Mamai-Gora for strontium isotope analysis. Dentition from the site of Bel’sk were analysed as part of a separate study [54]. The sampling strategy was designed to determine mobility over the lifetime of individuals from both sites. Dental calculus and adhering sediments were removed, and teeth were cleaned ultrasonically in ultra-pure water (deionized water). Teeth were then air-dried overnight and a very narrow slice of tooth enamel was removed along the vertical extent of the crown of the tooth with a circular diamond edged dental saw, with only the upper portion of the crown (occlusal surface) used for analysis. Sliced samples were ultrasonically cleaned in ultra-pure water (deionized water) and then dried at room temperature. The clean laboratory in the Department of Geological Sciences at the University of Cape Town was used to conduct strontium isotope analysis.

Tooth enamel samples were weighed into Teflon beakers and dissolved in 2 ml 65% two-bottle distilled HNO_3_, and then placed on a hotplate (140°C) for one hour. Samples were dried down and re-dissolved in 1.5 ml 2 M HNO_3_. Strontium separation chemistry followed methods discussed in [78]. After separation, the solutions for each sample were dried, dissolved in 2 ml 0.2% HNO_3_ and diluted to 200 ppb Sr concentrations for isotope analysis. Radiogenic ^87^Sr/^86^Sr ratios were measured using a Nu Instruments NuPlasma HR MC-ICP-MS. Analyses were referenced to the international Sr isotope standard with bracketing analyses of NIST SRM987 using a ^87^Sr/^86^Sr reference value of 0.710255 (following [79]). Instrumental mass fractionation was corrected using the exponential law, measured ^86^Sr/^88^Sr ratios and an accepted ^86^Sr/^88^Sr ratio of 0.1194 [80]. Isobaric interference of ^87^Rb on ^87^Sr was corrected using the measured ^85^Rb signal intensity and the natural ^85^Rb/^87^Rb ratio. The results for repeat analyses of an in-house carbonate reference material that were processed and measured with the batches of unknown samples analysed in this study gave an ^87^Sr/^86^Sr ratio of 0.708920 (2σ = 0.000030; n = 7) that agree with long-term results for this in-house reference material with an average ^87^Sr/^86^Sr ratio of 0.708911 (2σ = 0.000040; n = 414).

### Geologic substrates and strontium isotope ratio estimation

The mobility of humans in ancient environments is assessed through comparison with bioavailable ^87^Sr/^86^Sr ratios in soils, plants, water, and animals. Strontium isotope ratios of the plants and animals in a locale, and the humans consuming these resources, reflect the ^87^Sr/^86^Sr ratios of bedrock, soils, and water in that locale [81–84]. The small amount of strontium isotope variation that occurs as it is passed along the food chain is corrected during analysis [85–87]. We incorporated several different datasets to estimate ^87^Sr/^86^Sr ratios of bedrock geology and bioavailable strontium across Ukraine, which currently includes all bioavailable datasets for the region. Predictive modelling of ^87^Sr/^86^Sr ratios of various geological members across Europe [88], was compared to all published bioavailable ^87^Sr/^86^Sr ratios of ancient human enamel, rodent enamel, and plants [54,55,89], as well as values from ancient bison enamel [90]. Seven of the 13 archaeological sites under study had bioavailable strontium ratios from plants and/or fauna in the vicinity of the site (S3 Table in S1 File). For Scythian era sites we lack bioavailable Sr ratios for 5 of the 8 sites under study. However, comparisons of the predicted map based on pan-European modelling agrees well with published bioavailable data. Our map of geologic substrates has estimated ^87^Sr/^86^Sr ratios that compare well with existing plant values (between 0.001 and 0.0010 of the site average) and less so with some faunal values from the same locales (range of 0.0001 to 0.022 difference from site average) (S3 Table in S1 File).

Our map of geologic substrates is linked to predicted bioavailable ^87^Sr/^86^Sr ratios [88] and compares well with published ^87^Sr/^86^Sr ratios from ancient bison (*Bison priscus*) [90] as well as previously published ancient animal, soils, and human ratios [54,55] (Fig 2). The results of Voerkelius et al. demonstrate that by combining measured data (of groundwater) with a geologic map it is possible to predict qualitative spatial information of strontium isotope values (2010). Further, in geologically simple areas, Sr isotope ratios should be relatively homogeneous and mammal tissues can be accurately predicted [91]. Bedrock geology surrounding Vinogradnoe consists of Cenozoic substrates (0.7090 to 0.7110), with nearby Precambrian substrates (0.7120 to 0.7800) to the north and east. Similarly, the site of Peshtchanka is located in Cenozoic substrates, with nearby Precambrian substrates (0.7120 to 0.7800). Both the sites of Shakhta Stepnaya and Kirovograd are located within Cenozoic substrates, but have Precambrian substrates to their west, south, and east within 30 km. The sites of Alexandropol’, Ordzonikidze, Drana Kokhta, Zolotaya Balka, and Babina Mogila are located in Cenozoic substrates, but surrounded by pockets of Precambrian substrates. Near the site of Medvin, bedrock geology consists of Cenozoic gneiss, migmatite, and granite [92] with estimated strontium values ranging from 0.7090 to 0.7110. To the east of the site, Cenozoic substrates continue (0.7090 to 0.7110), while to the west and south Archean granitoids and Paleoproterozoic complexes [93] have predicted strontium values of 0.7120–0.7800. The bedrock geology in the vicinity of Mamai-Gora consists of Cenozoic substrates of gneiss, migmatite, and granite [92] with estimated strontium values ranging from 0.7090 to 0.7110. To the north (across the Dnieper River) and to the far east of the site are Precambrian substrates (crystalline and metamorphic rocks) [92] with predicted strontium values ranging from 0.7120 to 0.7800. In the vicinity of the Bel’sk complex, bedrock geology is Cenozoic with estimated strontium values ranging from 0.7090 to 0.7110 [88], while to the far north and east of the site are Mesozoic substrates, with estimated values from 0.7070 to 0.7090.

Local strontium isotope biosphere estimates were calculated for each site using the 3MAD_norm_ method, by identifying the median Sr ratio of ancient humans from the site ± triple the median absolute deviation (MAD) for humans (S3 Table in S1 File). The MAD is calculated by taking the absolute difference between each observed value and that of the sample set median. The MAD is scaled by multiplying this value by 1.4826 (following [94,95]). For each site the baseline reference was the median ±3MAD_norm_. This approach uses two robust estimators, as neither MAD nor the median are disproportionately impacted by extreme values [95]. The median absolute deviation (MAD) method has been proven to be a rigorous statistical method in the identification of outliers [95]. Overall, the average local range for the sites under study vary from ±0.0001 to ±0.020 and can be compared to previous strontium isotope studies worldwide with local ranges from 0.0010 to 0.0050 [96–98]. Distances of potential mobility for non-local individuals were determined using the map of geologic substrates (Fig 2). We measured from the site centre to the nearest substrate with similar estimated ^87^Sr/^86^Sr ratios to create a *minimum* distance of movement. For example, at sites where all individuals had ^87^Sr/^86^Sr ratios similar to geologic substrates, the radius around the site extended to the nearest geologic substrate with different ^87^Sr/^86^Sr ratios (Fig 2). Sites with individuals that had varied Sr ratios, from different substrates, had a radius that included a portion of that geologic substrate (Fig 2).

### Stable oxygen and carbon isotope values of ancient humans

We sampled 7 humans (12 teeth) from Medvin and 13 humans (26 teeth) from Mamai-Gora. Dentition from the site of Bel’sk were analysed as part of a separate study [54]. For oxygen and carbon isotope analyses, enamel samples were drilled from the cusp (occlusal surface) of the tooth as a point sample (bulk sample). Enamel samples were treated with 0.1 M acetic acid, mixed with a vortex and left to soak for 4 hours in order to remove diagenetic carbonates. Samples were then rinsed with deionized water, agitated, and then centrifuged; this was repeated a total of 5 times. After the final rinse, samples were frozen and freeze-dried. Carbonate samples were analysed for carbon (δ^13^C) and oxygen (δ^18^O) isotopes at the Leibniz Laboratory for Radiometric Dating and Stable Isotope Research at Christian Albrechts University of Kiel. Mass spectrometry was conducted on a Kiel IV carbonate preparation device connected to a MAT 253 mass spectrometer from ThermoScientific. During preparation the carbonates were reacted with 100% phosphoric acid (H_3_PO_4_) under vacuum at 75°C, and the evolved carbon dioxide was analysed eight times for each individual sample. All values are reported in the Vienna Pee Dee Bee notation (VPDB) relative to NBS19. Precision of all laboratory internal and international standards (NBS19 and IAEA-603) is <±0.05‰ for δ^13^C and <±0.09‰ for δ^18^O values. Error within a single tooth was <±0.07‰ for δ^13^C and <±0.06‰ for δ^18^O.

### Stable carbon and nitrogen isotope values of human bone collagen

We sampled the bones of 31 humans from Bel’sk, 15 humans from Mamai-Gora, and 12 humans from Medvin. For carbon and nitrogen isotope analyses, samples were collected from each individual with a preference for rib bones. Faunal material included 3 samples, two sheep and one pig. Bone was demineralized in 0.5M EDTA (pH 8.0) until collagen pseudomorphs were translucent and flexible. The resulting pseudomorphs were rinsed in distilled water seven times with an overnight soak to remove residual EDTA. The next day the samples were rinsed a further eight times with distilled water. The pseudomorphs were rinsed in 0.1 NaOH to remove humic acids, after which they were rinsed five more times in distilled water. Collagen samples were sent to the Boston University Stable Isotope Laboratory. Samples were analyzed using a EuroVector Euro EA elemental analyser coupled with a GVI IsoPrime in continuous flow mode. Analytical error was 0.1‰ and 0.2‰ for δ^13^C and δ^15^N, respectively.

Sample isotope ratios were compared to a secondary gas standard with an isotope ratio that was calibrated to international standards. For ^13^C_V-PDB_ the gas was calibrated against NBS 20 (Solnhofen limestone, −1.05 ± 0.02%). For ^15^N_air_ the gas was calibrated against atmospheric N_2_ and IAEA standards N-1, N-2 [(NH_4_)_2_SO_4_, 0.4 ± 0.2% and 20.3 ± 0.2% respectively], and NO3− (KNO_3_, 4.7 ± 0.2%). All international standards were obtained from the National Bureau of Standards in Gaithersburg, Maryland. Internal standards were measured repeatedly during the analysis and provided the following results: peptone (*n* = 4, δ^13^C = −14.77±0.05‰ and δ^15^N = 7.35±0.14‰); glycine (*n* = 4, δ^13^C = −33.97±0.08‰ and δ^15^N = 10.79 ± 0.13‰). The %C and %N values were calibrated against known quantities of the internal peptone and glycine standards. Isotopic values are reported in permil (‰) relative to the Vienna Pee Dee Belemnite (VPDB) standard for δ^13^C and atmospheric nitrogen (AIR) for δ^15^N. Collagen quality was assessed using %C, %N, and C:N ratios [99,100].

### Modern environmental and isotopic landscapes for carbon and oxygen isotope data

Climate and precipitation across Ukraine are discussed relative to the sites under study and the broader region. We have also included a discussion of terrestrial vegetation and the ecological zones that were inhabited. See Supplemental Text (S1 Text in S1 File)

### Statistical analysis

Isotopic (δ^13^C, δ^18^O) values of human enamel from different sites and periods were compared using an analysis of variance (ANOVA) paired with Tukey’s test in R [101]. Statistical analyses were used to determine the significance of differences between sites (separated by period) and between periods (all sites combined) (S2a-S2c Table in S1 File). In addition, a simple linear regression was performed on ^87^Sr/^86^Sr and δ^13^C values from the sites of Medvin and Mamai-Gora (S1 Fig and S4 Table in S1 File) to determine if there was a correlation between these two variables.

## Results

### Individual mobility at Scythian cemeteries

**Fig 3:**
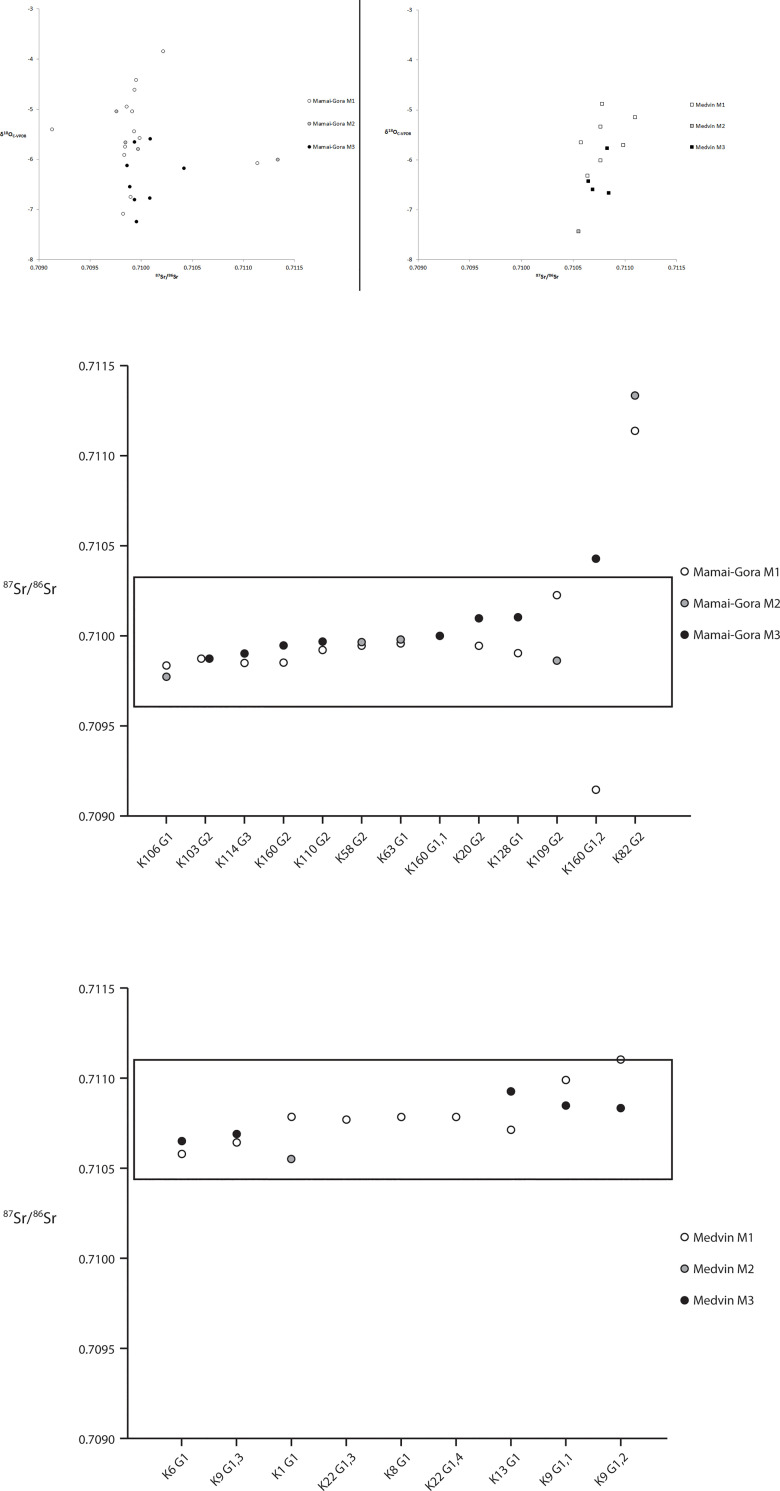
a. Correlation between stable oxygen isotope values and strontium isotope ratios of individuals from Mamai-Gora (left) and Medvin (right). b. Strontium isotope ratios for Mamai-Gora and Medvin (ascending plots, with baseline range).

We measured strontium isotope ratios for individuals from two cemeteries that have material culture associated with the Scythians. Both cemeteries are considered key sites with regard to understanding the mobility of Pontic steppe populations as they are well excavated and in different environmental zones. The results of our study, which measured ^87^Sr/^86^Sr ratios of human tooth enamel from the sites of Medvin (n = 15) and Mamai-Gora (n = 25), are shown in Fig 3 (Fig 3B; Tables 1 and 2). The ^87^Sr/^86^Sr ratios of human teeth from Medvin range from 0.7106 to 0.7111, with all teeth (15 of 15) sitting within the local range (0.7104 to 0.7111). Medvin is located on a Cenozoic substrate but surrounded by Palaeozoic substrates (0.7110 to 0.7130) and individuals had ^87^Sr/^86^Sr ratios (Figs 2, 3A and 3B) that suggest that they moved between these zones, with localized mobility being the most parsimonious explanation. Three individuals at Medvin (K1 G1; K13, G1; G9 G1,2) had slight differences in strontium isotope values between teeth of ≥0.0002, suggesting they may have engaged in short localized movements in the vicinity of the site. The ^87^Sr/^86^Sr ratios of human teeth analysed from Mamai-Gora range between 0.7091 and 0.7113, with the majority of teeth (21 of 25) falling within the local range estimated as 0.7096 to 0.7103 (Median Sr ratio ± 3MAD_norm_).

Based on modern geologic substrates, the site of Mamai-Gora is located on Cenozoic substrates, while Palaeozoic substrates with estimated ‘high’ strontium isotope values (0.7110–0.7130) are located along the northern bank of the Dnieper as well as ~130 km to the southeast. The closest geological locales with ‘low’ strontium values (<0.709) are located far to the northeast (~220 km away), in Crimea (~260 km), and west of Cherkasy (~280 km). At Mamai-Gora, one individual (K160 G1,2; Male) had an early forming molar (M1) exhibiting a low strontium value and a late forming molar (M3) with a high strontium value suggesting that they were born far outside of the region, then lived north or southeast of the site until adolescence, perhaps coming to live at Mamai-Gora after 16 years of age. A second individual (K82 G2; Female) had molars (M1, M2) with high strontium values suggesting that they lived in another region, either to the north or southeast of the site, and came to Mamai-Gora after adolescence. Three other individuals (K20 G2; K128 G1; K109 G2) exhibited smaller variation in strontium isotope values between teeth of ≥0.0002, suggesting they engaged in less restrictive forms of landscape use than individuals that had little variation between teeth.

A total of 38 teeth were analysed for human enamel δ^18^O _(C-VPDB)_ from the sites of Medvin (n = 12) and Mamai-Gora (n = 26) (Fig 3). δ^18^O values of human teeth from Medvin range between −4.9 and −7.4‰, with an average of −6.0‰, while δ^18^O values from Mamai-Gora range between −3.9 and −7.2‰, with an average of −5.8‰. At Medvin, all teeth fall within ±1.5‰ of the average value and at Mamai-Gora all teeth fall within ±1.5‰, except for one individual (#35; Female) with an M1 value of −3.9‰. The total range of oxygen isotope values for these two sites fall within the normal amount of variation (~3‰) for a population ([95,103–105]).

### Chronological variation in human mobility

**Fig 4:**
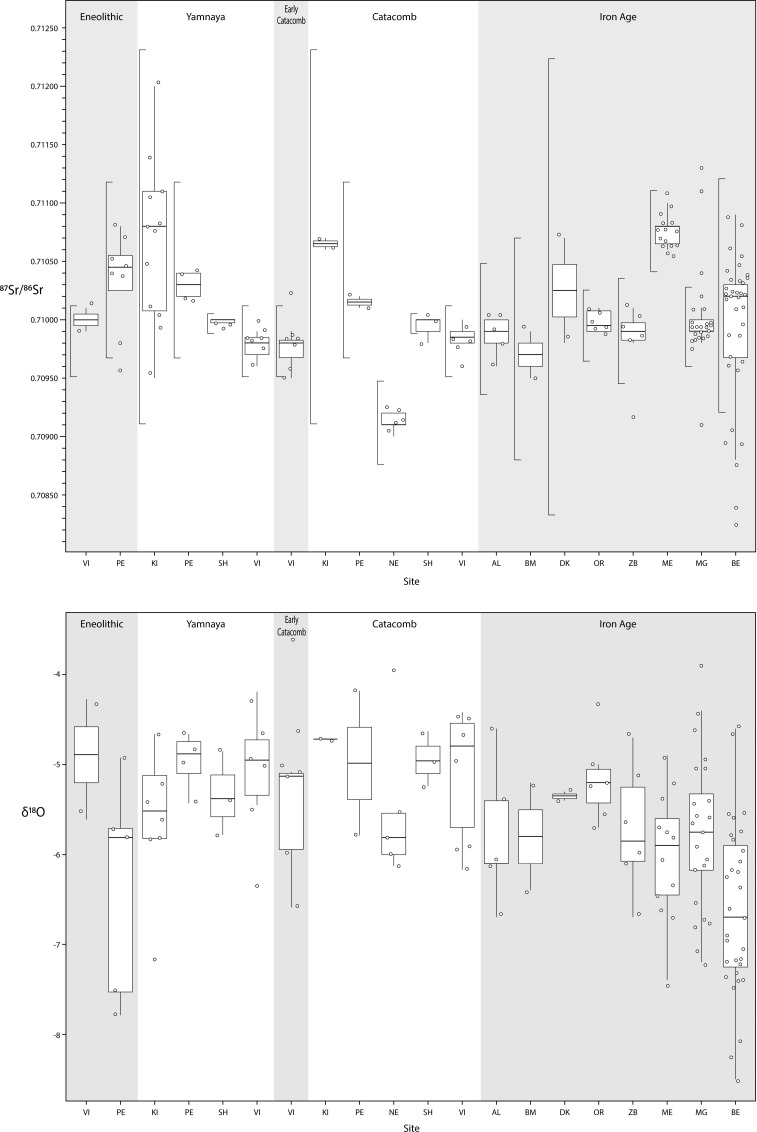
a. Boxplots of strontium isotope ratios for individuals across Ukraine, separated by site and period (bar to the left of each boxplot is the estimated local baseline). b. Boxplots of oxygen isotope ratios for individuals across Ukraine, separated by site and period. Site codes: Vinogradnoe (VI), Peshtchanka (PE), Kirovograd (KI), Shakhta Stepnaya (SH), Nevskoe (NE), Alexandropol (AL), Babina Mogila (BM), Drana Kokhta (DK), Ordzhonikidze (OR), Zolotaya Balka (ZB), Medvin (ME), Mamai-Gora (MG) and Bel’sk (BE).

We compared our newly measured ratios with published data we compiled from across Ukraine (Tables 1, 2; S1a-S1e and S3 Tables in S1 File) [54,55,102]. Sites from multiple chronological periods were included, from the Eneolithic to the Iron Age (Scythian era), permitting the study of shifting mobility strategies in Ukraine. The periods of study date to the Eneolithic (n = 2 sites; 3900–2900 cal. BCE) and Early Bronze Age Yamnaya (n = 4 sites; 3300–2500 cal BCE). There are also sites dating to the early Catacomb (2600–2200 cal BCE) and Catacomb (n = 5 sites; 2200–2000 cal BCE). Finally, there are several Iron Age (Scythian) sites (n = 8 sites) that date from 700–200 cal BCE) (Fig 4A). Strontium isotope values for individuals (n = 147) from the Eneolithic through the Iron Age unsurprisingly vary by site. Almost all of the sites under study (12 of 13) were located in an areas where distinct geolithologies are found within 25 km (Fig 2; S3 Table in S1 File). Only the site of Bel’sk was located in a region of Cenozoic substrates where the nearest variable lithology was over 90 km away.

Archaeological sites across Ukraine have consistently been identified in areas with Cenozoic substrates (0.7090–0.7110), except for the site of Nevskoe that is located within Mesozoic substrates (0.7070–0.7090) but in close proximity to Cenozoic substrates. Independent Sr reference datasets were not available for some of the sites under study (see S3 Table in S1 File), therefore we estimated baselines. Each site has a separate baseline (see Fig 4A; S3 Table in S1 File) that was calculated based on the median Sr ratio for the site ± triple the median absolute deviation or 3MAD_norm_ (see methods for discussion). We are confident in our estimation of the local biosphere as it takes into account variation between different sites located *within* Cenozoic substrates. The site of Vinogradnoe spans several periods (Eneolithic, Yamnaya, Early Catacomb, Catacomb), with an estimated baseline of 0.7098±0.0003 and three outliers. At Vinogradnoe, one outlier from the Eneolithic was an individual with a high value (0.7101) that fell just outside of our baseline, while in the Early Catacomb period, two individuals fell outside our estimated baseline, one with a high value (0.7102) and one with a low value (0.7095). The site of Peshtchanka (Eneolithic, Yamnaya, Catacomb) has an estimated baseline of 0.7104±0.0008, with one outlier with a low Sr ratio of 0.7096. Spanning both the Yamnaya and Catacomb periods, the site of Kirovograd has an estimated baseline of 0.7107±0.0016 with no outliers. A single outlier (0.7098) was identified at the site of Shakhta Stepnaya, falling just outside the estimated baseline of 0.7100±0.0001. The site of Nevskoe has an estimated baseline of 0.7091±0.0004, with no outlying individuals. Thus, there were five outlying individuals in total for Eneolithic, Early Catacomb, and Catacomb period sites, and no outlying individuals dating to Yamnaya period.

At Iron Age/Scythian era sites, the extent of mobility varied by site. At Alexandropol there were no outliers, with an estimated baseline of 0.7099±0.0006. At Babina Mogila and Drana Kokhta the estimated baselines were 0.7097±0.0010 and 0.7103±0.0020, respectively, with no outliers. The site of Ordzhonikidze had no outliers with an estimated baseline of 0.7100±0.0003. The site of Zolotaya Balka had a single outlier (O8; Male) with a low Sr ratio of 0.7092, well outside the baseline estimated at 0.7099±0.0005. At the site of Bel’sk, the local baseline was estimated as 0.7099±0.0010, with six outliers. Five individuals (2 M, 1 F; 2 Indet.) had low Sr ratios which are similar to areas located north or east of the site, at distances of 90 km and 160km respectively.

Stable oxygen isotope values from the Eneolithic through to the Iron Age vary by time period. In the Eneolithic, human δ^18^O values have a range from −4.3 to −7.8‰, similar to individuals from Yamnaya sites with a range from −4.2 to −7.2‰ [55]. Early Catacomb individuals had slightly higher oxygen isotope values (−3.6 to −6.6‰) that corresponded to values of later Catacomb era individuals (−4.0 to −6.2‰) [55]. Average δ^18^O values of human teeth from Scythian era sites overlap with these values, ranging from −3.9 to −7.2‰, except for the site of Bel’sk that had lower δ^18^O values ranging from −4.6 to −8.5‰ [54]. Significant differences in δ^18^O values (p<0.05, ANOVA paired with Tukey’s test) were identified between the site of Bel’sk and several other sites including Ordzhonikidze, Mamai-Gora, Vinogradnoe, and Peshtchanka (S2a Table in S1 File). Significant differences in average oxygen isotope values (p<0.05, ANOVA paired with Tukey’s test) were also identified between the Yamnaya and the Iron Age as well as between the Catacomb and the Iron Age (S2b Table in S1 File). The range of variation in δ^18^O values for a human community has been determined to be from 0.5 to 3.0‰ [95,103–105]. All sites have a range of values within 3‰, except for the sites of Bel’sk (4.0‰) and Peshtchanka (3.6‰). However, seasonal variation in oxygen isotopes of precipitation is extensive for Ukraine at c. 8‰, suggesting we might expect a wider range of human oxygen isotope values. Thus, the oxygen isotope data show no evidence for wide-ranging mobility in any periods, further supporting the Sr data.

### Range of diets among Scythian era populations

**Fig 5:**
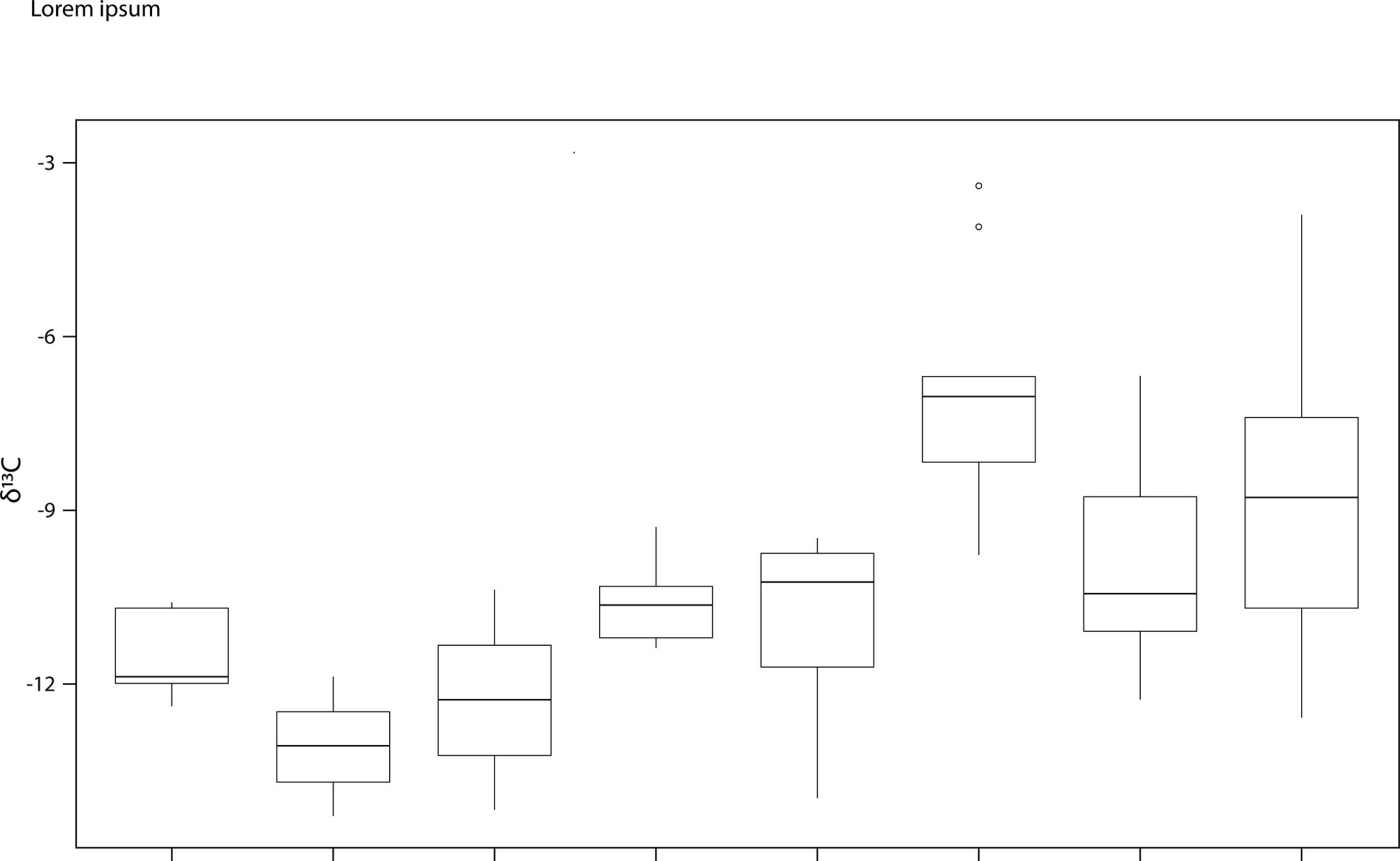
Boxplots of stable carbon isotope ratios for Iron Age/Scythian era individuals across Ukraine separated by site. Site codes: Vinogradnoe (VI), Peshtchanka (PE), Kirovograd (KI), Shakhta Stepnaya (SH), Nevskoe (NE), Alexandropol (AL), Babina Mogila (BM), Drana Kokhta (DK), Ordzhonikidze (OR), Zolotaya Balka (ZB), Medvin (ME), Mamai-Gora (MG) and Bel’sk (BE).

We analysed the same 38 teeth that were analyzed for δ^18^O for human enamel δ^13^C _(C-VPDB)_ at Medvin (n = 12) and Mamai-Gora (n = 26) (Fig 5). Stable carbon isotope values for individuals at Medvin ranged from −3.4 to −9.8‰ (average = −7.1‰), while δ^13^C values at Mamai-Gora ranged from −6.6 to −12.3‰ (average = −10.0‰). There is a significant difference between the mean values for these two cemeteries (p<0.05, ANOVA paired with Tukey’s test). At Medvin and Mamai-Gora, intra-individual variation ranged from 0.6 to 4.3‰, with an average difference between paired teeth of 2.4‰ and 1.6‰, respectively. Large differences in carbon isotope values between paired teeth were evident for some individuals, suggesting a shift in diet over time. For example, at Mamai-Gora one individual (#33) had an M1 measured at −12.0‰ and an M3 measured at −7.4‰ (difference = 4.6‰), while at Medvin there was an individual (#29) with an M1 measured at −7.1‰ and an M2 measured at −3.4‰ (difference = 3.8‰). Neither of these individuals exhibited differences in their strontium isotope ratios between teeth. The wide range of carbon isotope values of individuals at these sites suggests diversity in the amount of C_3_ and C_4_ plant consumption, or the consumption of livestock ingesting these plants. Enamel bioapatite δ^13^C values approaching −4.0‰ (dietary δ^13^C of approximately −13.0‰) align with those of individuals from regions of the globe with a documented reliance on C_4_ plants (e.g. [74]).

The range of δ^13^C for humans at Medvin (−3.4 to −9.8‰) and Bel’sk (−3.9 to −12.6‰) indicate a reliance on C_4_ food sources for at least some individuals. At Mamai-Gora, δ^13^C values for humans (−6.6 to −12.3‰), suggest more moderate consumption of C_4_ food sources. At the sites of Alexandropol, Ordzhonikidze, and Zolotaya Balka, δ^13^C values range from −9.3 to −14.0‰ [55] indicating very little consumption of C_4_ food sources. Finally, at the sites of Babina Mogila, Drana Kokhta, and Zolotaya Balka average values ranged from −10.4 to −14.3‰ [55] suggesting a reliance on C_3_ plants, or livestock ingesting C_3_ vegetation. Significant differences were identified between Medvin (p<0.05, ANOVA paired with Tukey’s test) and all other sites, except Bel’sk (S2c, S2e Table in S1 File). Unfortunately, no stable carbon isotope data were available for Eneolithic through Catacomb period sites.

A comparison of ^87^Sr/^86^Sr ratios and δ^13^C _(C-VPDB)_ values of human enamel indicates that there was no consistent correlation (strongly positive or negative) for the sites under study (S1 Fig in S1 File). Linear regression analysis demonstrates that for all sites (except Bel’sk) there was no significant relationship between strontium and carbon isotope values (S4 Table in S1 File). In addition, at all sites (including Bel’sk) less than 22% of samples fit the regression line (r^2^ ranged from 0.08 to 0.22) At Bel’sk there was a significant positive relationship (r = 0.41) between carbon and strontium values (p = 0.015).

**Fig 6:**
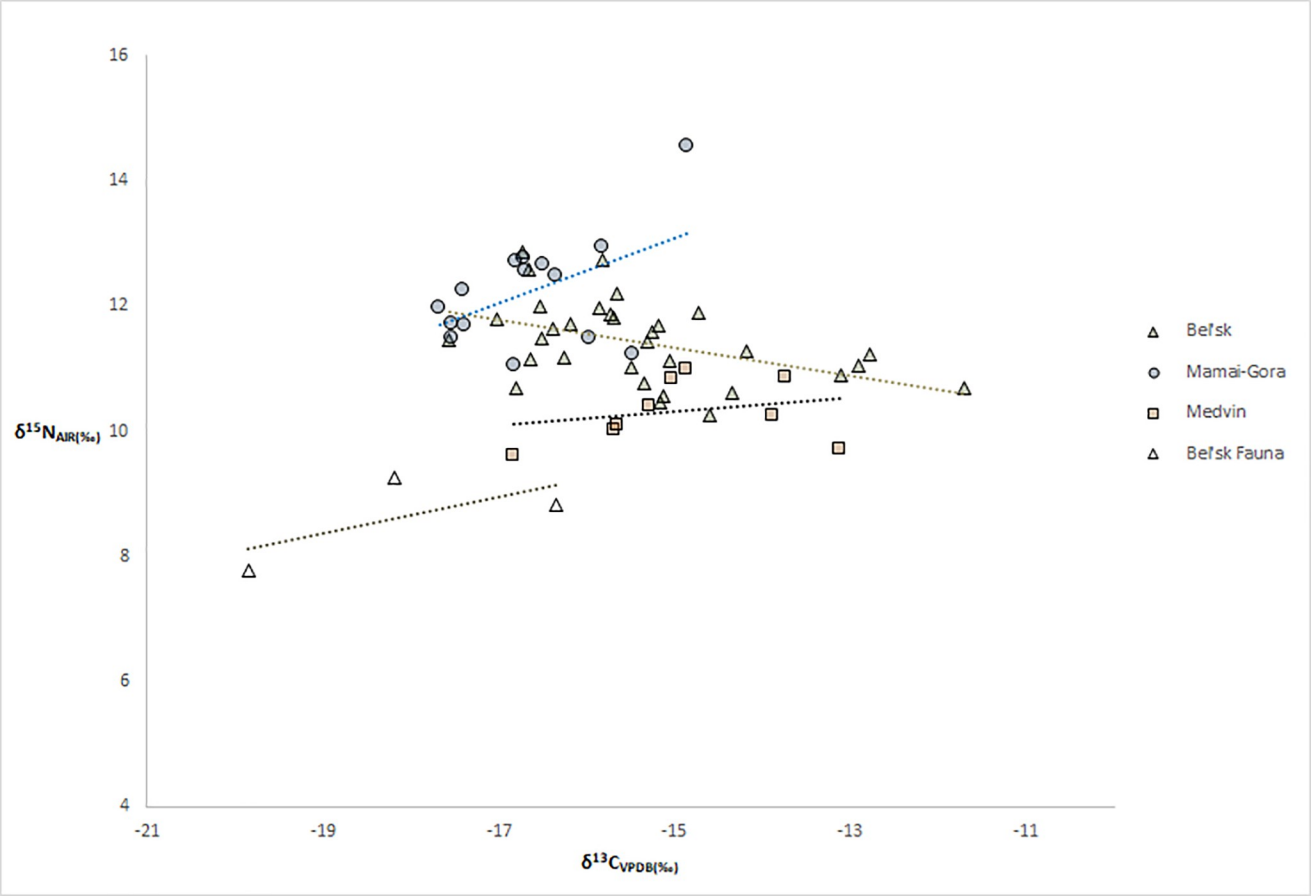
Plot of stable carbon and nitrogen isotope values of bone collagen from the sites of Bel’sk, Mamai-Gora and Medvin (all Scythian era).

We analysed a total of 58 human bone collagen samples from the sites of Bel’sk (n = 29), Mamai-Gora (n = 15), and Medvin (n = 10) (Fig 6; S1a, S1b, S1d Table in S1 File). Very few faunal remains were found in the collections, therefore we only analyzed only three samples (2 sheep, 1 pig). Stable carbon isotope values of individuals at Medvin ranged from −16.8 to −13.1‰ (average = −14.9‰), while δ^13^C values at Mamai-Gora were slightly higher, ranging from −17.7 to −14.9‰ (average = −16.6‰). At the site of Bel’sk, δ^13^C values of humans ranged from −17.6 to −12.8‰, with an average value of −15.5‰, while fauna (n = 3) had an average value of −18.1‰ (S1d Table in S1 File). Stable nitrogen isotope values of individuals from the site of Medvin ranged from 9.6 to 11.0‰ (average = 10.3‰), while the δ^15^N values at Mamai-Gora were approximately 2‰ higher with a range of 11.1 to 14.6‰ (average = 12.2‰). At Bel’sk, δ^15^N values of humans ranged from 10.3 to 12.9‰, with an average value of 11.4‰, while fauna (n = 3) had an average value of 8.6‰.

### Faunal and plant isotope references to understand human diet

The carbon isotopic values for animals at Bel’sk are contextualized within a discussion of available data on ancient faunal values, modern vegetation, and controlled studies. See Supplemental Text (S1 Text in S1 File)

## Discussion

### Mobility strategies among Scythian era populations

Our comparison of strontium isotope data (Figs 4A and 6) shows that there is very little isotopic evidence for group mobility in the Scythian era, indicating that life was less mobile than previously thought. This further supports previous research on human mobility in Ukraine [55,102], with additional datasets from Scythian era sites. Detailed mapping of geologic substrates, using estimated Sr values in the vicinity of each site, as published in [55], permitted investigation of patterns of mobility. While the majority of sites in this study were found on Cenozoic substrates, they also had diverse geologic formations nearby permitting some discussion of the extent of mobility (Fig 2). Our findings are supported by genetic evidence that demonstrates significant population interaction *prior* to the Iron Age in the region [21–24,34]. During the Eneolithic period, evidence suggests that individuals from the sites of Vinogradnoe and Peshtchanka engaged in localized mobility staying within Cenozoic substrates. Among Yamnaya populations, mobility was localized around each site, except for the site of Kirovograd, which lacked outliers but had at least one individual with a high Sr ratio similar to nearby Precambrian substrates. Overall, there was little evidence for long-distance mobility at any sites during the Early Catacomb and Late Catacomb periods [55,102]. By the Scythian epoch, there is strong evidence that some individuals engaged in inter-regional mobility. At Mamai-Gora, two individuals that were not local to the site may have arrived from within 25 km or from areas northeast of the site over 200 km away, while three local individuals likely engaged in landscape use within 25km of the site. Similarly, several individuals at Medvin may gave engaged in localized movements within 10km of the site. Five individuals from the Scythian era site of Bel’sk engaged in long-distance mobility from areas of Mesozoic substrates, the closest of which is 90 km away, while three other individuals could have come from southeastern areas as far as 160 km away. One individual at Zolotaya Balka had low Sr ratios that fell within those estimated for Cenozoic substrates, but also similar to individuals from Nevskoe where Mesozoic substrates are found. Our long chronological span provides good evidence that ‘non-local’ individuals from the Scythian era sites could have been coming from northern or eastern areas. However, they could also have also been traveling farther distances from the south (Crimea) or from far northwestern Ukraine.

Our findings suggest that, contrary to what has been suggested solely based on the archaeological record, many Scythian era individuals were not moving long distances (greater than 90 km). While a subset of populations apparently engaged in inter-regional mobility, there is also evidence for intensive localized mobility at some sites such as Bel’sk. One caveat is that at some sites there is a lack of variation in geological substrates across a wide area, thus masking smaller-scale local and regional movement. Scythian era populations across Eurasia were engaging in pastoralism and horseback riding, drawing on this component of their lifestyle to develop shared motifs, while simultaneously engaging in agricultural production [106,107]. Highly mobile Scythian era individuals were seemingly travelling much longer distances than in previous periods. As urban centers grew, an influx of populations would have made trade vital, underpinning the movement of goods during the Scythian epoch [8]. In concert, migratory events predating the Iron Age indicate that many urban centers were culturally diverse focal points and that early mobility set the stage for the networks that followed. We argue that the Scythian epoch was clearly a period of contradictions, with strong evidence for complex interactions between agro-pastoralists and pastoralists that contributed to population aggregation in urban locales.

### Scythian diets: A complex Iron Age steppe

In addition to this direct assessment of individual mobility, evidence for variability in diets among Iron Age populations has highlighted the complexity of Scythian lifeways. Floral biomes in Ukraine support mainly C_3_ vegetation [108,109], thus any evidence of C_4_ sources in human or animal diets are likely from the consumption of domesticated crops, namely millet. Observed δ^13^C values for faunal bone collagen at Bel’sk range from −19.8 to −16.3‰, indicating a mixed diet of C_3_ vegetation as well as small amounts of C_4_ plants such as millet. Among societies practicing pastoralism (lacking domesticated grains) isotopic data demonstrate a shared carbon isospace of humans and livestock, from the same region and time period [76]. However, humans and fauna at the Iron Age sites under study share very little carbon isospace indicating that diets included a partial reliance on foodstuffs that were not typical for Pontic steppe floral biomes, such as domesticated millet. At Bel’sk, populations had varied access to millet as a staple product, while those moving to the site later in life did not consume as much millet [76]. The population at Medvin had diverse diets, with an overall moderate amount of millet consumption that was on average higher than for individuals from Mamai-Gora, where fish may have played a larger part in diets. Findings from Mamai-Gora indicate that two individuals engaging in inter-regional mobility consumed less millet than other people, while another individual engaged in regional movement also consumed less millet.

The fact that individuals that were highly mobile apparently consumed lower levels of millet may suggest that their diets were based mainly on pastoral products or C_3_ plants. In contrast, individuals with low levels of mobility had a wider spectrum of dietary intake varying from high levels of millet consumption, to diets based almost exclusively on pastoral products or C_3_ plants. Linear regression analysis demonstrates that for all sites (except Bel’sk) there was no significant relationship between strontium and carbon isotope values, nor was there a consistent positive or negative correlation (S1 Fig; S4 Table in S1 File). Evidence of *in situ* millet cultivation from nearby demonstrates that agriculture was an important subsistence strategy during this period [16,17]. The results of this study lend further support for agriculture being a significant part of Scythian era economies in both the steppe and forest-steppe. Our findings suggest a reassessment of lifeways during the Scythian epoch, from highly mobile pastoralists to diverse urbanites engaging in a wide range of subsistence strategies, which indicate that these globalized societies had different subsets or groups with varied economic strategies [16].

## Conclusions

Romantic perceptions of nomadic Scythians focus on the extent of mobility, gene-flow between populations, and engagements in warfare [3] that are grounded in evidence from burial assemblages, including weapons, armaments, and other personal accoutrements. This discourse engages with approaches that identify broad similarities in material culture that shroud important information on urbanization, human movement, and subsistence economies. Current genetic evidence highlights gene flow between past populations writ large but does not address individual mobility within local geographies. Direct evidence for diet and mobility provides an archive of past diet and residence that offers an alternative perspective on Scythian lifeways. Our results indicate that inter-regional mobility was limited during the Scythian era, yet likely higher than in previous periods. Millet became an important dietary staple among many urbanites. High dietary diversity suggests that urban locales were key nodes of socio-economic integration that may have included individuals engaged in varied economic endeavours (e.g. pastoralism, agriculture). It is clear that if we are to truly uncover the ‘Scythians’ we need to accept that the Eurasian steppe was home to a myriad of dynamic cultures and subsistence strategies during the Iron Age. In fact, it is perhaps variability, rather than a uniformity of nomadic warriors, that truly frames the Scythians as predecessors to incipient globalization in Eurasia.

Our study highlights the potential of using isotopic analysis to directly assess prevailing models of Scythian mobility. Mobility varied widely among and between communities, with evidence that some individuals participated in wider, long-distance networks during this period. Yet, there is also evidence for localized mobility and less mobile populations. Future work in the region with larger sample sizes that encompass multi-generational populations should be able to provide further insights into human mobility between site types (urban centers versus rural settings), as well as between individuals with different grave goods and apparent social status. More detailed primary mapping work will enable a greater understanding of isotopic variation across space in this understudied region (for discussion see [110]). Although these undertakings are often expensive and time consuming, they provide robust support for the exploration of archaeological hypotheses at a higher level of resolution. For example, oxygen isotope analysis of surface waters and modern animals in several seasons could be explored in relation to intra-tooth analysis of humans from the same locale in order to examine seasonal environmental changes versus mobility. In addition, variation in the bioavailable strontium across the landscape, using Sr of contemporary plants and small mammals, can be mapped at multiple scales to clarify the extent of human movement. In this way, we can move further away from assumed stereotypes of migration and nomadism towards dynamic, context-specific insights into what it meant to be ‘Scythian’.

## Supporting information

S1 File (PDF)
