# Supplementary material for: Re-evaluating Scythian lifeways: Isotopic analysis of diet and mobility in Iron Age Ukraine
Source: PLoS One. 2021 Mar 10;16(3):e0245996. doi: 10.1371/journal.pone.0245996 (PMC7946291; doi:10.1371/journal.pone.0245996)
Supplement: S1 File — (PDF) [file pone.0245996.s001.pdf]

Re-evaluating Scythian lifeways: Isotopic analysis of diet and mobility in Iron Age Ukraine

A. R. Ventresca Miller[1,2,3,4*], J. Johnson[5], S. Makhortykh[6], C. Gerling[7], L. Litvinova[6], S. Andrukh[8], G. Toschev[8], J. Zech[1], P. le Roux[9], C. Makarewicz[3,4], N. Boivin[1,10,11,12], P. Roberts[1,10]

*Corresponding author, ventrescamiller@shh.mpg.de, avenmil@umich.edu

[1] Max Planck Institute for the Science of Human History, Department of Archaeology, Stable Isotope Group, Kahlaische Strasse 10, 07745 Jena, Germany

[2] Department of Anthropology and Museum of Anthropological Archaeology, University of Michigan, Ann Arbor 48109, Michigan, United States

[3] Graduate School of Human Development in Landscapes, Kiel University, Leibnizstrasse 3, 24118 Kiel, Germany

[4] Institute for Prehistoric and Protohistoric Archaeology, Kiel University, Johanna-Mestorf-Strasse 2-6, 24118 Kiel, Germany

[5] Department of Anthropology, University of Wyoming, 12th and Lewis Streets, Laramie, Wyoming 82070, United States

[6] Institute of Archaeology of National Academy of Sciences Ukraine (NUAS), 12 av. Geroyiv Stalingrada, 04210 Kyiv, Ukraine

[7] Integrative Prehistory and Archaeological Science, Department of Environmental Sciences, University of Basel, Spalenring 145, 4055 Basel, Switzerland

[8] Zaporizhzhya National University, Soborniy Avenue 74, 69002 Zaporizhzhya, Ukraine

[9] Department of Geological Sciences, University of Cape Town, Rondebosch, 7700, South Africa

[10] School of Social Science, University of Queensland, Brisbane, Australia

[11] Department of Archaeology, University of Calgary, Calgary, Canada

[12] Smithsonian Institution, New York, USA

Radiocarbon dating

We sampled four human bones for radiocarbon dating, which were run at the Poznan Radiocarbon Laboratory (S5 Table). Collagen degradation in bone was checked by measuring the content of nitrogen and carbon in bone, using analyser Flash EA 1112 Series (ThermoScientific), with suitable samples having nitrogen in bone above 0.6%, and C/N ratio not higher than 5. Bones were crushed mechanically, and powder was treated with 2M HCl at room temperature for 20 minutes, then with 0.1M NaOH for one hour. After each step of treatment, the sample was centrifuged, and residuum was collected. Extraction of collagen is processed in HCl (pH=3, 80°C, 10h), and after centrifugation, residuum was removed. Extracted collagen was then ultrafiltered on pre-cleaned Vivaspin 15 MWCO 30 kD filters [1]. Quality of the collagen is ultimately assessed basing on C/N atomic ratio (interval of acceptance: 2.7-3.5) and collagen extraction yield (acceptance threshold:

0.5%). Content of ^{14}C in a sample of carbon is measured using the spectrometer "Compact Carbon AMS" (produced by: National Electrostatics Corporation, USA) as described [2]. The measurement is performed by comparing intensities of ionic beams of ^{14}C, ^{13}C and ^{12}C measured for each sample and for standard samples (modern standard: “Oxalic Acid II” and standard of ^{14}C-free carbon: “background”). In each AMS run, 30-33 samples of unknown age are measured, alternated with measurements of 3-4 samples of modern standard and 1-2 samples of background.

The site of Mamai-Gora was previously dated from the end of the 5th to the 2rd century BCE based on detailed analyses of material culture from burials [3–5]. Similarly, analyses of material culture from Medvin allows for the separation of burials into two chronological groups, which date from the 7th to 6th centuries BC, and from the 5th to 4th centuries BCE [6–8]. Our current radiocarbon dating of human occipital bones (kurgan 125, grave 1; kurgan 128, grave 1) from Mamai-Gora gave conventional radiocarbon ages of 2265 ± 30 BP (Poz-93203) and 2195 ± 35 BP (Poz-93205). Calibrated date ranges (IntCal13) are from 399-209 cal BC and 369-174 cal BCE (OxCal ver 4.2.3) [9,10]. Recently published radiocarbon dates from burials at Medvin [11] support these findings with conventional radiocarbon ages of 2455 ± 30 BP (kurgan 6, Grave 1, 1) and 2500 ± 30 BP; calibrated date ranges (IntCat13) from 756-413 cal BC and 788-537 cal BC, respectively. At Medvin we radiocarbon dated a human occipital (kurgan 8, grave 1) and a human sacrum (kurgan 6, grave 1) that had conventional radiocarbon ages of 2480 ± 35 BP (Poz-9320) and 2320 ± 35 BP (Poz-93202), with calibrated date ranges from 775-431 cal BCE and 484-233 cal BCE. Slightly earlier radiocarbon dates are not unexpected, as other dating programs have similarly pushed relative dates back several hundred years [12,13].

Modern environmental and isotopic landscapes for carbon and oxygen isotope data

The climate across Ukraine is continental with moderately cold winters and comparatively warm summers. Medvin (182 masl) is located along a small tributary of the Ros’ river, within the broader watershed and floodplain of this larger river system, in the Kyiv’ska region where temperatures range from −6° C in the winter to 26° C in the summer. Mamai-Gora (57 masl) is located in the Zaporizhia region with temperatures in the summer reaching 22° C and as low as −3° C in the winter. The site is situated along the southern (left) bank of the Dnieper River that is designated as part of the Dnieper lowlands, an extended valley within a system of floodplains [14]. The Dnieper originates in the Smolensk region of Russia and eventually flows into the Black Sea, with tens of thousands of tributaries feeding the river along the way. Currently, the Dnieper River has multiple dams that have altered its formerly wide floodplain. This navigable waterway is an important transport route today as it likely was in prehistory [15,16].

In central Ukraine, there is a high degree of seasonal variation in $\delta^{18}O$ values of precipitation [17]. There are very few water stations in Ukraine. We focused on three that were located in the northeast at Kharkiv, in the east at Svyatohirs’k, and far to the west at L’viv. At the Karkhiv water station (152 masl) the average $\delta^{18}O$ value of meteoric water is −7.4‰, with values ranging from a seasonal low of −12.4‰ in March to a high in July of −4.2‰ [17]. The average temperature ranges from −6.9 to 19°C. The Svyatohirs’k station (163 masl) recorded an average $\delta^{18}O$ value for meteoric water of −9.2‰, with values ranging from a low of −13.2‰ in January to −5.5‰ in July. At the L’viv station (296 masl) values vary in $\delta^{18}O$ from −14.9‰ in February to −7.6‰ in August,

with temperatures ranging from −3.5 to 17.7°C respectively. Only a single locale has published surface water $\delta^{18}O$ values, which are from the Seversky Donets River near Kharkiv and range from −6.2 to −11.7‰ from May and September, respectively. Nearby, groundwater $\delta^{18}O$ values range from −10.1 to −12.5‰ for the same period [18,19].

The direct comparison of ancient $\delta^{18}O_{dw(VSMOW)}$ values and modern precipitation values is not recommended as the transformation of human $\delta^{18}O_{c(VPDB)}$ to drinking water values $\delta^{18}O_{dw(VSMOW)}$ include large error ranges of up to 3.5‰ [20–22]. Furthermore, the extent of environmental change is unknown for many regions, leading to inaccurate interpretations of mobility. Therefore, we have not transformed human $\delta^{18}O_{c(VPDB)}$ to drinking water values $\delta^{18}O_{dw(VSMOW)}$ instead comparing a range of values for each site. At a single locale, the range of variation in human $\delta^{18}O$ values has been determined as 0.5 to 3.0‰ [23–26]. Seasonal variation in stable oxygen isotope measurements of precipitation for the region, based on modern measured values, is extensive at *c.* 8.0‰, suggesting that in strongly seasonal environments we might expect a wider range of human $\delta^{18}O$ values for individuals who resided in a single locale during tissue formation. Accounting for strong seasonal variation, we calculated the local range for individuals drinking from the same water source as the median value ±1.5‰ [26].

We investigated the stable carbon isotope measurements of terrestrial vegetation in the vicinity of the sites of Medvin and Mamai-Gora to inform the dietary intake of Scythian era populations. Modern environmental zones are on a north-south gradient shifting from mixed forests, to forest-steppe, to open steppe vegetation in the south, with the Black Sea littoral along the southern border (Fig 1). The site of Medvin is located in central Ukraine within the forest-steppe zone (49°24'26.9"N 30°50'45.0"E). This region is characterized by deciduous woodlands interspersed with meadow steppe, while to the north there are mixed coniferous and deciduous forests [27,28]. Heading south, the forests gradually decrease in density and open steppe predominates. Mamai-Gora (47°26'07.7"N 34°16'31.1"E) is located just south of the Dnieper River in the open steppe. Central and southern Ukraine consists of highland and lowland plains, with floral biomes supporting mainly C_3 vegetation. Terrestrial plants using a C_3 pathway exhibit average $\delta^{13}C$ values of −27‰, while those following a C_4 pathway have higher average $\delta^{13}C$ values of −14‰ [29].

To clarify human enamel apatite $\delta^{13}C$ values, we determined the best offset between enamel apatite and diet, which varies by taxa and by tissue type. The offset between diet and apatite is ~14.0‰ for herbivore bone carbonate and ~9.5‰ for carnivore bone carbonate [30–33]. For human enamel apatite, an offset of ~9.0‰ has been suggested to be appropriate [31,34–36]. We use the following equation from Ambrose and Norr (1993): $\delta^{13}C_{diet} = 1.04 * \delta^{13}C_{apatite} - 9.2$ for all humans in this study. Average herbivore $\delta^{13}C$ values (of enamel) for 100% C_3 and C_4 reliance in pre-industrial CO_2 conditions are approximately −12.0‰ and 0.0‰, respectively [37,38]. Tooth enamel isotope values of humans with a 100% reliance on C_3 plants are estimated at −13.0 and −17.0‰, while humans with a high reliance on C_4 plants are estimated at −5.0 to −1.0‰ [30], however, it should be borne in mind that a lack of experimental work means these values are poorly defined for humans.

Faunal and plant isotope references to understand human diet

The carbon isotopic values for animals at Bel'sk range from −19.8 to −16.3‰, indicating a mixed diet of both C_3 and C_4 plants, therefore, it is possible that livestock at Bel'sk ingested small to

moderate amounts of C_4 plants such as millet that were being farmed in the vicinity of the site. The observed range of $\delta^{13}C$ for humans at Medvin (−16.8 to −13.1‰), Mamai-Gora (−17.7 to −14.9‰) and Bel'sk (−17.6 to −12.8‰) only overlap slightly with the faunal values, with the majority exceeding them more than the expected 1‰ trophic increase. The average faunal $\delta^{13}C$ values of −18.6‰, were 3.1‰ lower than the average human values of Bel'sk (−15.5‰). This suggests that the dietary intake of humans relied partially on livestock and C_3 plants, alongside C_4 food sources such as millet. Measurements of modern millets (in controlled environments and across Eurasia) produce a range of $\delta^{13}C$ values from −10.0 to −14.1‰ and $\delta^{15}N$ values that range from 1.8 to 7.8‰ [39–42]. Assuming a diet-consumer enrichment of 3-5‰, the range of human collagen $\delta^{15}N$ values for humans (9.6 to 14.6‰) fits well within the adjusted range of values (+3-5‰) for both modern millets (4.8 to 12.8‰) and ancient herbivores from Bel'sk (10.8 to 14.3‰). When plotted together, $\delta^{13}C$ and $\delta^{15}N$ measurements of bone collagen suggest that millet was an important part of dietary intake in the Scythian era. In addition, slightly higher $\delta^{15}N$ values at the site of Mamai-Gora might indicate that fish were a dietary component.

We identified a similar pattern in the carbon and nitrogen isotopic composition of humans and livestock from the Pontic steppe as was previously discussed in Ventresca Miller and Makarewicz [43] for much of Central and Inner Asia. At Bel'sk there is a positive correlation between $\delta^{13}C$ and $\delta^{15}N$ values for livestock, but a breakdown in this correlation for humans, suggesting a departure from Pontic steppe dietomes. Livestock with a positive correlation between $\delta^{13}C$ and $\delta^{15}N$ values could have ingested small amounts of naturally available C_4 plants or arid impacted C_3 vegetation, rather than feeding on fallow fields. At the sites of Bel'sk, Medvin and Mamai-Gora there was a shift in the average $\delta^{13}C$ values relative to reference livestock. Pastoral societies (lacking domesticated grains) have isotopic data that demonstrate a shared carbon isospace between humans and livestock, from the same region and time period [43]. As humans and fauna at the Iron Age sites under study share very little carbon isospace, their dietary intake included a partial reliance on foodstuffs atypical for Pontic steppe floral biomes, such as domesticated millet.

References

1. Bronk Ramsey C, Higham T, Bowles A, Hedges R. Improvements to the pretreatment of bone at Oxford. Radiocarbon. 2004;46: 155–163.

2. Goslar T, Czernik J, Goslar E. Low-energy 14C AMS in Poznań radiocarbon laboratory, Poland. Nuclear instruments and methods in physics research section B: Beam Interactions with Materials and Atoms. 2004;223: 5–11.

3. Andrukh SI. Mamai-Gora cemetery. Zaporizhia: Zaporizhzhya National University; 2001.

4. Andrukh SI, Toschev GN. Mamai-Gora cemetery. Zaporizhia: Zaporizhzhya National University; 1999.

5. Andrukh SI, Toschev GN. Mamai-Gora burial ground of the Scythian time. In: Wojciech B, editor. Peregrinationes archaeologicae in Asia et Europa Joanni Chochorowski dedicatae, Wydawnictwo Profil-Archeo. Krakow; 2012. pp. 485–490.

6. Kovpanenko GT. The kurgans of the Scythian time near Medvin in Ross-river basin. Scythians and Sarmatians. Kyiv; 1977. pp. 40–72.

7. Levchenko BM. The excavaton of the first kurgan group near Medvin-village in the Boguslav region (by the materials of excavations 1982, 1984—1985). In: Grechko DS, Shelekhan AB, editors. Grishkovsky cemetery of the Scythians in Kharkov region. Kyiv; 2012. pp. 109–139.

8. Levchenko BM, Levchenko NB, Grechko DS. The barrows of the early Scythian time near Medvin in Ross-river basin (by the materials of excavations 1984—1985). Archaeology and ancient history of Ukraine. 2015;2: 202–218.

9. Bronk Ramsey C. Methods for Summarizing Radiocarbon Datasets. Radiocarbon. 2017; 1–25.

10. Reimer PJ, Bard E, Bayliss A, Beck JW, Blackwell PG, Ramsey CB, et al. IntCal13 and Marine13 radiocarbon age calibration curves 0–50,000 years cal BP. Radiocarbon. 2013;55: 1869–1887.

11. Järve M, Saag L, Scheib CL, Pathak AK, Montinaro F, Pagani L, et al. Shifts in the Genetic Landscape of the Western Eurasian Steppe Associated with the Beginning and End of the Scythian Dominance. Current Biology. 2019;29: 2430-2441. e10.

12. Hanks BK, Epimakhov A, Renfrew A. Towards a refined chronology for the Bronze Age of the southern Urals, Russia. Antiquity. 2007;81: 353–367.

13. Beisenov AZ, Svyatko SV, Kassenalin AE, Zhambulatov KA, Duisenbai D, Reimer PJ. First radiocarbon chronology for the Early Iron Age sites of Central Kazakhstan (Tasmola culture and Korgantas period). Radiocarbon. 2016;58: 179–191.

14. Физико-географическое районирование Украинской ССР. Киев: Киевский университет; 1968.

15. Dewdney JC. A Geography of the Soviet Union: Pergamon Oxford Geographies. Elsevier; 2013.

16. Samoilenko Y. The principle of freedom of international river navigation and problems of regime of navigational usage of international rivers, which flow through the territory of Ukrainian State. Proceedings of Ukrainian scientific and practical conference "Actual problems of contemporary international law"(Sevastopol, Ukraine, 21-23 June 2012). 2012.

17. IAEA/WMO. In: Global network of isotopes in precipitation, The GNIP Database [Internet]. Available: http://www.iaea.org/ water

18. Vystavna Y, Diadin D, Huneau F. Describing stable water isotopes framework for the surface water, groundwater and precipitation in East Ukraine. 7th International Conference on Water Resources and Environment Research, Kyoto TERRSA, Kyoto. 2016. pp. 5–9.

19. Vystavna Y, Diadin D, Huneau F. Defining a stable water isotope framework for isotope hydrology application in a large trans-boundary watershed (Russian Federation/Ukraine).

Isotopes in Environmental and Health Studies. 2018;54: 147–167. doi:10.1080/10256016.2017.1346635

20. Pollard AM, Pellegrini M, Lee-Thorp JA. Some observations on the conversion of dental enamel δ18Op values to δ18Ow to determine human mobility. American Journal of Physical Anthropology. 2011;145: 499–504.

21. Pryor AJ, Stevens RE, O'Connell TC, Lister JR. Quantification and propagation of errors when converting vertebrate biomineral oxygen isotope data to temperature for palaeoclimate reconstruction. Palaeogeography, Palaeoclimatology, Palaeoecology. 2014;412: 99–107.

22. Ventresca Miller AR. Modeling modern surface water δ18O to explore prehistoric human mobility. In: Ventresca Miller, Makarewicz CA, editors. Isotopic Investigations of Pastoralism in Prehistory. New York: Routledge; 2018. pp. 41–56.

23. White C, Longstaffe FJ, Law KR. Exploring the effects of environment, physiology and diet on oxygen isotope ratios in ancient Nubian bones and teeth. Journal of Archaeological Science. 2004;31: 233–250.

24. Ehleringer JR, Bowen GJ, Chesson LA, West AG, Podlesak DW, Cerling TE. Hydrogen and oxygen isotope ratios in human hair are related to geography. Proceedings of the National Academy of Sciences. 2008;105: 2788–2793.

25. Daux V, Lécuyer C, Héran M-A, Amiot R, Simon L, Fourel F, et al. Oxygen isotope fractionation between human phosphate and water revisited. Journal of human evolution. 2008;55: 1138–1147.

26. Lightfoot E, O'Connell TC. On the use of biomineral oxygen isotope data to identify human migrants in the archaeological record: intra-sample variation, statistical methods and geographical considerations. PloS one. 2016;11: 0153850.

27. Dolukhavnov PM. Geography of East European Plain. In: Dolukhanov PM, Sarson GR, Shukurov AM, editors. The East European plain on the eve of agriculture. Archaeopress; 2009. pp. 9–15.

28. Sorokina L, Doroshkevich S, Kushnir A. Ancient and modern landscapes of Bels'k settlement as a human habitats. Ukrainian Geographical Magazine. 2014;3: 25–33.

29. O'Leary MH. Carbon isotope fractionation in plants. Phytochemistry. 1981;20: 553–567.

30. Krueger HW, Sullivan CH. Models for Carbon Isotope Fractionation Between Diet and Bone. Stable Isotopes in Nutrition. American Chemical Society; 1984. pp. 205–220. doi:10.1021/bk-1984-0258.ch014

31. Ambrose SH, Norr L. Experimental evidence for the relationship of the carbon isotope ratios of whole diet and dietary protein to those of bone collagen and carbonate. Prehistoric human bone. Springer; 1993. pp. 1–37.

32. Koch PL, Fogel ML, Tuross N. Tracing the diets of fossil animals using stable isotopes. In: Lajtha K, Michener B, editors. Stable Isotopes in Ecology and Environmental Science. Boston: Blackwell Scientific Publication; 1994. pp. 63–92.

33. Cerling TE, Harris JM. Carbon isotope fractionation between diet and bioapatite in ungulate mammals and implications for ecological and paleoecological studies. Oecologia. 1999;120: 347–363.

34. Ambrose SH, Butler BM, Hanson DB, Hunter-Anderson RL, Krueger HW. Stable isotopic analysis of human diet in the Marianas Archipelago, Western Pacific. American Journal of Physical Anthropology. 1997;104: 343–361. doi:10.1002/(SICI)1096-8644(199711)104:3<343::AID-AJPA5>3.0.CO;2-W

35. Krigbaum J. Neolithic subsistence patterns in northern Borneo reconstructed with stable carbon isotopes of enamel. Journal of Anthropological Archaeology. 2003;22: 292–304. doi:10.1016/S0278-4165(03)00041-2

36. Kellner CM, Schoeninger MJ. A simple carbon isotope model for reconstructing prehistoric human diet. American Journal of Physical Anthropology. 2007;133: 1112–1127.

37. Lee-Thorp JA, Sealy JC, Van Der Merwe NJ. Stable carbon isotope ratio differences between bone collagen and bone apatite, and their relationship to diet. Journal of archaeological science. 1989;16: 585–599.

38. Levin NE, Simpson SW, Quade J, Cerling TE, Frost SR. Herbivore enamel carbon isotopic composition and the environmental context of Ardipithecus at Gona, Ethiopia. Geol Soc Am Spec Pap. 2008;446: 215–234.

39. McGovern PE, Zhang J, Tang J, Zhang Z, Hall GR, Moreau RA, et al. Fermented beverages of pre-and proto-historic China. Proceedings of the National Academy of Sciences. 2004;101: 17593–17598.

40. Pechenkina EA, Ambrose SH, Xiaolin M, Benfer RA. Reconstructing northern Chinese Neolithic subsistence practices by isotopic analysis. Journal of Archaeological Science. 2005;32: 1176–1189.

41. Hu Y, Wang S, Luan F, Wang C, Richards MP. Stable isotope analysis of humans from Xiaojingshan site: implications for understanding the origin of millet agriculture in China. Journal of Archaeological Science. 2008;35: 2960–2965. doi:10.1016/j.jas.2008.06.002

42. Lightfoot E, Przelomska N, Craven M, O′Connell TC, He L, Hunt HV, et al. Intraspecific carbon and nitrogen isotopic variability in foxtail millet (*Setaria italica*): Carbon and nitrogen isotopic variability in foxtail millet. Rapid Commun Mass Spectrom. 2016;30: 1475–1487. doi:10.1002/rcm.7583

43. Ventresca Miller A, Makarewicz CA. Intensification in pastoralist cereal use coincides with the expansion of trans-regional networks in the Eurasian Steppe. Sci Rep. 2019; 8363.

Sample Number	Individual Number	Cemetery	Grave	Individual	# of paired teeth	Sex	Age Range	$^{87}/^{86}Sr$ (M1)	$^{87}/^{86}Sr$ (M2)	$^{87}/^{86}Sr$ (M3)	Sr difference between paired teeth	$\delta^{18}O_{(C-VDPB)}$ (M1)	$\delta^{18}O_{(C-VDPB)}$ (M2)	$\delta^{18}O_{(C-VDPB)}$ (M3)	$\delta^{18}O$ difference between paired teeth	$\delta^{13}C_{(C-VDPB)}$ (M1)	$\delta^{13}C_{(C-VDPB)}$ (M2)	$\delta^{13}C_{(C-VDPB)}$ (M3)	$\delta^{13}C$ difference between paired teeth	$\delta^{15}N$ (collagen)	$\delta^{13}C$ (collagen)
223	23	Medvin	K 8, Grave 1	1 of 1	–	Female	> 40 yrs		–	0.7108	–	–	–	–	–	–	–	–	–	10.6	-13.9
225, 226	24	Medvin	K-13, grave 1	1 of 1	2	Male	> 50 yrs	0.7107	–	0.7109	0.0002	–	–	–	–	–	–	–	–	–	–
228, 229	25	Medvin	K 6, Grave 1	1 of 1	2	Male	40 - 50 yrs	0.7106	–	0.7106	0.0001	-5.7	–	-6.4	0.8	-6.7	–	-4.1	2.6	10.8	-13.1
231, 232	26	Medvin	K 9, Grave 1	1 of 3	2	Male	24 - 35 yrs	0.7110	–	0.7108	0.0001	-5.7	–	-6.7	1.0	-7.3	–	-9.1	1.8	9.7	-14.9
234, 235	27	Medvin	K 9, Grave 1	2 of 3	2	Indet.	35 - 40 yrs	0.7111	–	0.7108	0.0003	-5.2	–	-5.8	0.6	-7.0	–	-9.8	2.8	10.3	-16.8
237, 238	28	Medvin	K 9, Grave 1,	3 of 3	2	Indet.	30 - 35 yrs	0.7106	–	0.7107	0.0000	-6.3	–	-6.6	0.3	-8.0	–	-6.8	1.2	9.6	-15.7
240, 241	29	Medvin	K 1, Grave 1	1 of 1	2	Male	> 30 yrs	0.7108	0.7106	–	0.0002	-4.9	-7.4	–	2.6	-7.1	-3.4	–	3.8	11.0	-13.7
242	–	Medvin	K 22, Grave 1	2 of 6	–	Male	> 45 yrs	–	–	–	–	–	–	–	–	–	–	–	–	10.9	-15.6
243	–	Medvin	K 22, Grave 1	1 of 6	–	Male	> 55 yrs	–	–	–	–	–	–	–	–	–	–	–	–	10.1	-14.6
246	30	Medvin	K 22, Grave 1	4 of 6	–	Subadult	6 - 10 yrs	0.7108	–	–	–	-5.3	–	–	–	-8.7	–	–	–	10.0	-15.3
248	31	Medvin	K 22, Grave 1	3 of 6	–	Subadult	4 -8 yrs	0.7108	–	–	–	-6.0	–	–	–	-6.7	–	–	–	10.4	-15.0
**Average**								0.7108	0.7106	0.7108	**0.0002**	-5.6	-7.4	-6.4	**1.0**	-7.4	-3.4	-7.4	**2.4**	10.3	-14.9

S1a Table. Isotope values for individuals from the site of Medvin (ME)

Sample Number	Individual Number	Cemetery	Grave	Individual	# of paired teeth	Sex	Age Range	$^{87}/^{86}Sr$ (M1)	$^{87}/^{86}Sr$ (M2)	$^{87}/^{86}Sr$ (M3)	Sr difference between paired teeth	$\delta^{18}O_{(C\text{-}VDPB)}$ (M1)	$\delta^{18}O_{(C\text{-}VDPB)}$ (M2)	$\delta^{18}O_{(C\text{-}VDPB)}$ (M3)	$\delta^{18}O$ difference between paired teeth	$\delta^{13}C_{(C\text{-}VDPB)}$ (M1)	$\delta^{13}C_{(C\text{-}VDPB)}$ (M2)	$\delta^{13}C_{(C\text{-}VDPB)}$ (M3)	$\delta^{13}C$ difference between paired teeth	$\delta^{15}N$ (collagen)	$\delta^{13}C$ (collagen)
174, 176	32	Mamai-Gora	K. 110, Grave 2	1 of 1	2	Male	17 - 40 yrs	0.7099	–	0.7100	0.0000	-5.0	–	-7.2	2.2	-11.7	–	-11.0	0.7	12.5	-16.3
183	–	Mamai-Gora	K. 125	1 of 1	–	Male	28 - 55 yrs	–	–	–	–	–	–	–	–	–	–	–	–	12.5	-16.3
185, 186	33	Mamai-Gora	K. 114, Grave 3	1 of 1	2	Male	20 - 35 yrs	0.7098	–	0.7099	0.0001	-5.9	–	-6.6	0.6	-12.0	–	-7.4	4.6	11.2	-15.5
188, 189	34	Mamai-Gora	K. 106, Grave 1	1 of 1	2	Male	40 - 50 yrs	0.7098	0.7098	–	0.0001	-7.1	-5.0	–	2.0	-8.1	-10.7	–	2.6	–	–
191, 192	35	Mamai-Gora	K. 109, Grave 2	1 of 1	2	Female	30 - 45 yrs	0.7102	0.7099	–	0.0004	-3.9	-5.7	–	1.8	-11.0	-6.7	–	4.3	12.7	-16.5
194, 195	36	Mamai-Gora	K. 103, Grave 1	1 of 1	2	Female	24 - 30 yrs	0.7099	–	0.7099	0.0000	-5.0	–	-6.1	1.2	-9.0	–	-9.5	0.5	12.0	-17.7
197, 198	37	Mamai-Gora	K. 160, Grave 1	1 of 2	2	Subadult	3.5 - 6.5 yrs	0.7100	–	–	–	-5.6	-5.3	–	0.3	-8.6	-10.2	–	1.6	14.6	-14.9
200, 201	38	Mamai-Gora	K. 160, Grave 1	2 of 2	2	Male	21 - 35 yrs	0.7091	–	0.7104	0.0013	-5.4	–	-6.2	0.8	-10.1	–	-11.2	1.1	12.3	-17.4
203, 204	39	Mamai-Gora	K. 160, Grave 2	1 of 1	2	Female	15 - 23 yrs	0.7098	–	0.7099	0.0001	-5.8	–	-6.8	1.1	-7.5	–	-8.7	1.2	11.5	-16.0
206, 207	40	Mamai-Gora	K. 63, Grave 1	1 of 1	2	Male	>35 yrs	0.7100	0.7100	–	0.0000	-4.4	-5.8	–	1.4	-7.3	-11.1	–	3.8	12.9	-15.8
209, 210	41	Mamai-Gora	K. 20, Grave 2	1 of 1	2	Male	30 - 40 yrs	0.7099	–	0.7101	0.0002	-5.4	–	-6.8	1.3	-9.2	–	-10.9	1.8	11.1	-16.8
211	–	Mamai-Gora	K. 132, Grave 1	1 of 1	–	Male	30 - 40 yrs	–	–	–	–	–	–	–	–	–	–	–	–	11.7	-17.4
213, 214	42	Mamai-Gora	K. 128, Grave 1	1 of 1	2	Male	20 - 40 yrs	0.7099	–	0.7101	0.0002	-6.8	–	-5.6	1.2	-12.2	–	-11.6	0.5	11.7	-17.5
215	–	Mamai-Gora	K. 153, Grave 1	1 of 1	–	Male	40 - 50 yrs	–	–	–	–	–	–	–	–	–	–	–	–	12.7	-16.8
216, 217	43	Mamai-Gora	K. 82, Grave 2	1 of 1	2	Female	35 - 45 yrs	0.7111	0.7113	–	0.0002	-6.1	-6.0	–	0.1	-12.3	-10.9	–	1.4	11.5	-17.5
220, 221	44	Mamai-Gora	K. 58, Grave 2	1 of 1	2	Male	40 - 50 yrs	0.7100	–	0.7099	0.0000	-4.6	–	-5.7	1.0	-9.8	–	-11.1	1.3	12.6	-16.7
**Average**								0.7100	0.7102	0.7100	**0.0002**	-5.5	-5.6	-6.4	**1.2**	-9.9	-9.9	-10.2	**2.0**	12.2	-16.6

S1b Table. Human isotope values for the Iron Age site of Mamai-Gora (MG)

Sample Number	Individual Number	Cemetery	Grave	Sex	Age Range	$^{87/86}Sr$ (M1)	$^{87/86}Sr$ (M2)	$^{87/86}Sr$ (M3)	Sr difference between paired teeth	$\delta^{18}O_{(C-VDPB)}$ (M1)	$\delta^{18}O_{(C-VDPB)}$ (M2)	$\delta^{18}O_{(C-VDPB)}$ (M3)	$\delta^{18}O$ difference between paired teeth	$\delta^{13}C_{(C-VDPB)}$ (M1)	$\delta^{13}C_{(C-VDPB)}$ (M2)	$\delta^{13}C_{(C-VDPB)}$ (M3)	$\delta^{13}C$ difference between paired teeth	Reference
AL 1	N/A	Alexandropol	K. 5, Grave 1	Male	20-30 years			0.7100	–			-6.1	–			-10.7	–	Gerling 2015
AL 2	N/A	Alexandropol	K. 12, Grave 4	Male	25-30 years	0.7099			–	-4.6			–	-12.4			–	Gerling 2015
AL 3	N/A	Alexandropol	K. 15, Grave 6	Male	17-22 years	0.7100			–	-6.1			–	-12.0			–	Gerling 2015
AL 4	N/A	Alexandropol	K. 15, Grave 8	Male	45-55 years			0.7096	–			-6.7	–			-10.6	–	Gerling 2015
AL 5	N/A	Alexandropol	K. 10, Grave 3	Male	25-35 years			0.7098	–			-5.4	–			-11.9	–	Gerling 2015
O1	N/A	Babina Mogila	burial chamber 1	Male	old adult	0.7099			–	-5.2			–	-11.9			–	Gerling 2015
O3	N/A	Babina Mogila	burial chamber 3	Subadult	13-15 years	0.7095			–	-6.4			–	-14.3			–	Gerling 2015
O4	N/A	Drana Kokhta	niche 5	Subadult	14-15 years			0.7107	–			-5.3	–			-14.2	–	Gerling 2015
O16	N/A	Drana Kokhta	central burial	Male	50-55 years			0.7098	–			-5.4	–			-10.4	–	Gerling 2015
O15	N/A	Ordzhonikidze	K. 15, Grave 1	Male	30-40 years		0.7101		–		-5.5		–		-10.3		–	Gerling 2015
O12	N/A	Ordzhonikidze	K. 15, Grave 2	Female	40-45 years			0.7101	–			-5.2	–			-10.4	–	Gerling 2015
O9	N/A	Ordzhonikidze	K. 32, Grave 4 (F)	Female	50-60 years		0.7099		–		-4.3		–		-9.3		–	Gerling 2015
O14	N/A	Ordzhonikidze	K. 32, Grave 4 (M)	Male	50-60 years			0.7100	–			-5.2	–			-11.3	–	Gerling 2015
O11	N/A	Ordzhonikidze	K. 33, Grave 1	Male	40-50 years		0.7099		–		-5.0		–		-11.4		–	Gerling 2015
O13	N/A	Ordzhonikidze	K. 33, Grave 3	Female	40-50 years			0.7099	–			-5.7	–			-10.9	–	Gerling 2015
O7	N/A	Zolotaya Balka	K. 13, Grave 1	Indet.		0.7101			–	-5.1			–	-12.2			–	Gerling 2015
O2	N/A	Zolotaya Balka	K. 15, Grave 1 (Adult)	Female	22-25 years	0.7098			–	-4.7			–	-10.3			–	Gerling 2015
O5	N/A	Zolotaya Balka	K. 15, Grave 1 (Adolescent)	Indet.	12-13 years (15-17)	0.7099			–	-6.1			–	-14.0			–	Gerling 2015

O8	N/A	Zolotaya Balka	K.15, Grave 2	Male	45-55 years			0.7092	–			-6.0	–			-9.6	–	Gerling 2015
O10	N/A	Zolotaya Balka	K. 15, Grave 3	Female	20-22 years		0.7099		–		-6.7		–		-9.5		–	Gerling 2015
O6	N/A	Zolotaya Balka	K. 22, Grave 1	Female	old adult ?			0.7100	–			-5.7	–			-10.2	–	Gerling 2015
**Average**						0.7099	0.7099	0.7099	–	-5.5	-5.4	-5.7	–	-12.4	-10.1	-11.0	–	

S1c Table. Isotope values for individuals from the Iron Age sites of Alexandropol (AL), Babina Mogila (BM), Drana Kokhta (DK), Ordzhonikidze (OR), and Zolotaya Balka (ZB)

Sample Number	Individual Number	Cemetery	Grave	Individual	# of paired teeth	Sex	Age Range	$^{87}/^{86}Sr$ (M1)	$^{87}/^{86}Sr$ (M2)	$^{87}/^{86}Sr$ (M3)	Sr difference between paired teeth	$\delta^{18}O$ (C-VDPB) (M1)	$\delta^{18}O$ (C-VDPB) (M2)	$\delta^{18}O$ (C-VDPB) (M3)	$\delta^{18}O$ difference between paired teeth	$\delta^{13}C$ (C-VDPB) (M1)	$\delta^{13}C$ (C-VDPB) (M2)	$\delta^{13}C$ (C-VDPB) (M3)	$\delta^{13}C$ difference between paired teeth	$\delta^{15}N$ (collagen)	$\delta^{13}C$ (collagen)
100	–	Pereschepino	K-22	1 of 4	–	Subadult	9 - 15 yrs	–	–	–	–	–	–	–	–	–	–	–	–	11.3	-14.2
104	1	Pereschepino	K-22	2 of 4	2	Male	45 - 55 yrs	0.7084	–	0.7082	0.0001	-5.5	–	-6.3	0.7	-10.0	–	-10.1	0.099	11.9	-14.7
105	2	Pereschepino	K-22	3 of 4	–	Indet.	>21 yrs	–	–	0.7103	–	–	–	-7.4	–	–	–	-7.7	–	–	–
107	3	Pereschepino	K-22	4 of 4	2	Subadult	12 - 18 yrs	0.7104	–	0.7108	0.0004	-4.6	–	-5.8	1.3	-9.9	–	-5.8	4.056	–	–
110	–	Pereschepino	K-15	1 of 2	–	Indet.	Indet.	–	–	–	–	–	–	–	–	–	–	–	–	11.3	-14.2
111	4	Pereschepino	K-15	2 of 2	2	Subadult	12 - 18 yrs	0.7106	–	0.7102	0.0004	-5.6	–	-7.0	1.4	-8.0	–	-8.6	0.636	12.9	-16.7
114	–	Pereschepino	K-19	1 of 2	–	Indet.	35 - 55 yrs	–	–	–	–	–	–	–	–	–	–	–	–	12.2	-15.7
121	5	Pereschepino	K-21	1 of 2	2	Subadult	7 - 11 yrs	0.7109	0.7105	–	0.0003	-6.7	-8.3	–	1.6	-5.8	-3.9	–	1.876	11.0	-12.9
124	6	Pereschepino	K-21	2 of 2	–	Male	19 - 48 yrs	–	–	0.7102	–	–	–	-7.4	–	–	–	-8.6	–	12.0	-15.8
125	–	Pereschepino	K-20	1 of 2	–	Male	40 - 44 yrs	–	–	–	–	–	–	–	–	–	–	–	–	10.9	-13.1
126	–	Pereschepino	K-20	2 of 2	–	Indet.	Indet.	–	–	–	–	–	–	–	–	–	–	–	–	11.9	-15.7
128	–	Pereschepino	K-23	2 of 2	–	Indet.	Indet.	–	–	–	–	–	–	–	–	–	–	–	–	10.3	-14.6
129	7	Pereschepino	K-23	1 of 2	2	Male	35 - 50 yrs	0.7102	–	0.7103	0.0001	-6.1	–	-7.2	1.1	-6.6	–	-7.0	0.397	11.1	-15.1
131	–	5th Field	K-1	1 of 1	–	Indet.	Adult	–	–	–	–	–	–	–	–	–	–	–	–	11.8	-17.0
134	8	Cemetery Б	K-4	1 of 3	2	Male	24 - 55 yrs	0.7100	–	0.7096	0.0003	-6.0	–	-5.8	0.2	-11.0	–	-7.9	3.0265	–	–
136	–	Pereschepino	K-22	1b of 4	–	Subadult	>13 yrs	–	–	–	–	–	–	–	–	–	–	–	–	12.6	-16.7
138	9	Cemetery Б	K-4	2 of 3	2	Subadult	>15 yrs	–	0.7097	0.7096	0.0001	–	-6.2	-6.6	0.4		-12.2	-9.0	9.0	10.8	-15.3
141	10	Cemetery Б	K-4	3 of 3	–	Subadult	8.5 - 11.5 yrs	–	0.7101	–	–	–	-5.5	–	–	–	-12.5	–	–	11.2	-16.6
147	11	Cemetery Б	K-3	1 of 1	–	Indet.	30 - 35 yrs	–	–	0.7099	–	–	–	-7.4	–	–	–	-8.2	–	11.7	-16.2
148	–	Tsarina	Ash mound 1, Altar 2	2 of 3	–	Female	>35 yrs	–	–	–	–	–	–	–	–	–	–	–	–	10.6	-15.1
149	–	Tsarina	Ash mound 1, Altar 2	3 of 3	–	Male	Adult	–	–	–	–	–	–	–	–	–	–	–	–	11.0	-15.5
172	12	Tsarina	Ash mound 1, square L-02, Shtyke 3	1 of 1	–	Subadult	6 - 10 yrs	0.7105	–	–	–	-7.3	–	–	–	-7.8	–	–	–	11.6	-15.3
144	13	Tsarina	Ash mound 1 altar 2	1 of 3	2	Male	>40 yrs	0.7101	–	0.7102	0.0001	Failed	–	-6.4	–	Failed	–	-10.0	–	–	–
157	14	Tsarina	Ash mound 3, square A-2, sht. 3	1 of 1	2	Male	20 - 35 yrs	0.7102	–	0.7089	0.0013	-5.7	–	-6.9	1.2	-12.6	–	-11.4	1.2	10.7	-16.8

159	–	Tsarina	Ash mound 3, Altar 3, sk. 2		–	Subadult	>13 yrs	–	–	–	–	–	–	–	–	–	–	–	–	11.7	-15.2
160	15	Tsarina	Ash mound 3, skeleton 1 (by tag on bag)		2	Indet.	40 - 50 yrs	0.7088	–	0.7096	0.0008	-4.6	–	-6.2	1.5	-7.3	–	-10.9	3.6	–	–
164	16	Tsarina	Ash mound 3, A-24, Altar		2	Female	>21 yrs	–	0.7104	0.7104	0.0000	–	-8.1	-7.2	0.9	–	-7.1	-9.3	2.2	10.5	-15.2
166	17	Tsarina	Altar skeleton 1		2	Female	20 - 24 yrs	0.7091	–	0.7102	0.0012	-7.1	–	-7.5	0.4	-11.0	–	-9.9	1.0	–	–
168	–	Tsarina	Ash mound 1, square L-02, Shtyke 3		–	Indet.	Adult	–	–	–	–	–	–	–	–	–	–	–	–	11.6	-16.4
169	–	Tsarina	Square Zh.1, Shtyke 5		–	Indet.	Indet.	–	–	–	–	–	–	–	–	–	–	–	–	11.4	-15.3
151	18	Field 8	Kurgan-2	0 of 2	2	Male	>45 yrs	0.7102	0.7102	–	0.0000	-7.2	-8.5	–	1.3	-8.4	-11.0	–	2.6	11.5	-16.5
153	–	Field 8	Kurgan-2	1 of 2	–	Indet.	Adult	–	–	–	–	–	–	–	–	–	–	–	–	12.0	-16.5
154	–	Field 8	Kurgan-2	2 of 2	–	Indet.	Adult	–	–	–	–	–	–	–	–	–	–	–	–	11.5	-17.6
178	19	Osnyagi 3rd Field	K1, Sq. T-5, 0.9-1.2m	1	2	Male	>45 yrs	0.7103	0.7103	–	0.0000	-7.2	-7.2	–	0.0	-7.1	-6.5	–	0.6	11.2	-12.8
181	20	Marchenki 9th Field	K1, N. 2, Sq. IK-18, 1.2 m	2 of 2	2	Indet.	> 45 yrs	0.7089	0.7099	–	0.0009	-5.8	-6.5	–	0.6	-9.6	-12.3	–	2.6	11.2	-16.2
146	21	Cemetery Б	K-3	Rodent	–	Indet.	30 - 35 yrs	0.7102	–	–	–	–	–	–	–	–	–	–	–	–	–
**Average**								0.7	0.7102	0.7	**0.0004**	-6.1	-7.2	-6.8	**0.9**	-8.9	-9.4	-8.9	**2.2**	11.4	-15.5
101		Pereschepino	K-19	Fauna (Sheep)	–			–	–	–	–	–	–	–	–	–	–	–	–	7.8	-19.8
132		5th Field	K-1	Fauna (Sheep)	–			–	–	–	–	–	–	–	–	–	–	–	–	9.3	-18.2
120		Pereschepino	K-21	Fauna (Pig?)	–			–	–	–	–	–	–	–	–	–	–	–	–	8.8	-16.3
**Average**																				8.6	-18.1

S1d Table. Isotope values for humans and fauna from the Iron Age site of Bel'sk (BE)

Sample Number	Time Period	Cemetery	Grave	Individual	# of paired teeth	Sex	Age Range	$^{87/86}Sr$ (M1)	$^{87/86}Sr$ (M2)	$^{87/86}Sr$ (M3)	Sr difference between paired teeth	$\delta^{18}O_{(C\text{-}VDPB)}$ (M1)	$\delta^{18}O_{(C\text{-}VDPB)}$ (M2)	$\delta^{18}O_{(C\text{-}VDPB)}$ (M3)	$\delta^{18}O$ difference between paired teeth	Reference
UK 7, 46	Yamnaya	Kirovograd	5		2	Male	25-35 years	0.7101	0.7100		0.0001		-5.4		–	Gerling 2015
UK 8, 43	Yamnaya	Kirovograd	10		2	Subadult	8-11 years	0.7111	0.7120		0.0009	-4.9	-5.8		0.97	Gerling 2015
UK 44, 45	Yamnaya	Kirovograd	10		2	Male	35-45 years		0.7114	0.7111	0.0003		-5.2		–	Gerling 2015
SU 11	Yamnaya	Kirovograd	14		–	Male	30-35 years	0.7108			–	-4.7			–	Gerling 2015
SU 10	Yamnaya	Kirovograd	16		–	Male	45-50 years		0.7099		–		-7.2		–	Gerling 2015
UK 41, 42	Yamnaya	Kirovograd	20		–	Male	45-55 years		0.7105	0.7095	0.0009		-5.8		–	Gerling 2015
UK 9, 47	Yamnaya	Kirovograd	24		–	Subadult	15-18 years	0.7108	0.7108		0.0001		-5.6		–	Gerling 2015
UK 48, 49	Catacomb	Kirovograd	13		–	Male	35-50 years	0.7106	0.7107		0.0001	-4.7	-4.7		0.01	Gerling 2015
UK 38, 33	Eneolithic	Peshtchanka	Kurgan 1, Burial 6		2	Male	45-50 years	0.7108		0.7104	0.0005			-5.7	–	Gerling 2015
UK 39, 34	Eneolithic	Peshtchanka	Kurgan 1, Burial 7		2	Male	30-40 years		0.7104	0.7107	0.0003			-4.9	–	Gerling 2015
UK 4, 36	Eneolithic	Peshtchanka	Kurgan 1, Burial 14		2	Male	35-40 years	0.7098	0.7096		0.0002	-7.8	-7.5		0.26	Gerling 2015
UK 2, 37	Eneolithic	Peshtchanka	Kurgan 1, Burial 15		2	Female	30-40 years	0.7105	0.7105		0.0001		-5.8		–	Gerling 2015
UK 1, 40	Yamnaya	Peshtchanka	Kurgan 1, Burial 10		2	Male	25-30 years	0.7102		0.7102	0.0000	-5.0		-4.7	0.33	Gerling 2015
UK 10, 35	Yamnaya	Peshtchanka	Kurgan 1, Burial 11		2	Subadult	11-13 years	0.7104	0.7104		0.0000	-4.8	-5.4		0.66	Gerling 2015
UK 58, 57	Catacomb	Peshtchanka	Kurgan 1, Burial 12		2	Indet.	Indet.	0.7102	0.7101		0.0001	-4.2	-5.8		1.61	Gerling 2015
UK 51, 52	Yamnaya	Shakhta Stepnaya	Kurgan 2, Burial 7		2	Male	35-40 years	0.7099		0.7100	0.0000	-5.8			–	Gerling 2015
UK 53, 54	Yamnaya	Shakhta Stepnaya	Kurgan 4, Burial 11		2	Indet.	40-45 years		0.7100	0.7100	0.0000		-5.4	-4.9	0.53	Gerling 2015

UK 50	Catacomb	Shakhta Stepnaya	Kurgan 2, Burial 4		–	Female	25-35 years	0.7098			–	-4.6			–	Gerling 2015
UK 55, 56	Catacomb	Shakhta Stepnaya	Kurgan 4, Burial 10		2	Female	20-25 years	0.7100		0.7100	0.0000	-5.0		-5.2	0.28	Gerling 2015
UK 30	Eneolithic	Vinogradnoe	Kurgan 3, Burial 15		–	Male	35-40 years		0.7099		–		-5.5		–	Gerling 2015
UK 14	Eneolithic	Vinogradnoe	Kurgan 24, Burial 30			Male	40-50 years			0.7101	–			-4.3	–	Gerling 2015
UK 27, 31	Yamnaya	Vinogradnoe	Kurgan 3, Burial 25		2	Male	~ 25 years		0.7099	0.7098	0.0001		-4.9	-6.4	1.46	Gerling 2015
UK 3, 22	Yamnaya	Vinogradnoe	Kurgan 24, Burial 8	1	2	Indet. (Male?)	23-25 years	0.7098	0.7098		0.0001		-5.0		–	Gerling 2015
UK 11, 12	Yamnaya	Vinogradnoe	Kurgan 24, Burial 8	2	2	Subadult	11-14 years	0.7096	0.7096		0.0000	-4.2	-4.7		0.48	Gerling 2015
UK 21	Yamnaya	Vinogradnoe	Kurgan 24, Burial 31			Male	30-45 years			0.7099	–			-5.5	–	Gerling 2015
UK 28	Early Catacomb	Vinogradnoe	Kurgan 24, Burial 15	1	–	Male	35-45 years			0.7098	–			-6.6	–	Gerling 2015
UK 15, 23	Early Catacomb	Vinogradnoe	Kurgan 24, Burial 15	2	2	Female	30-45 years		0.7096	0.7095	0.0001			-5.1	–	Gerling 2015
UK 13	Early Catacomb	Vinogradnoe	Kurgan 24, Burial 19	1		Male	25-35 years			0.7102	–			-5.1	–	Gerling 2015
UK 6, 16	Early Catacomb	Vinogradnoe	Kurgan 24, Burial 22		2	Male	17-20 years	0.7098		0.7099	0.0000	-3.6		-5.1	1.46	Gerling 2015
UK 24	Early Catacomb	Vinogradnoe	Kurgan 24, Burial 34	1		Female	30-35 years			0.7098	–			-6.0	–	Gerling 2015
UK 19	Early Catacomb	Vinogradnoe	Kurgan 24, Burial 34	2		Male	30-35 years			0.7097	–			-5.9	–	Gerling 2015
UK 20, 18	Catacomb	Vinogradnoe	Kurgan 24, Burial 17	1	2	Male	45-55 years	0.7100	0.7098		0.0002	-4.7	-6.0		1.27	Gerling 2015
UK 32, 29	Catacomb	Vinogradnoe	Kurgan 3, Burial 36		2	Indet. (Female?)	50-55 years	0.7099	0.7099		0.0000	-4.4	-4.9		0.48	Gerling 2015
UK 17, 25	Catacomb	Vinogradnoe	Kurgan 24, Burial 26		2	Male	30-40 years	0.7098		0.7096	0.0001			-6.2	–	Gerling 2015
UK 5, 26	Catacomb	Vinogradnoe	Kurgan 24, Burial 32		2	Male	40-45 years	0.7099	0.7098		0.0001	-4.5			–	Gerling 2015
OU 2	Catacomb	Nevskoe	Kurgan 2, Burial 10			Indet.	Adult			0.7092	–			-6.0	–	Gerling 2015

OU 1	Catacomb	Nevskoe	Kurgan 3, Burial 3			Indet.	Adult	0.7090			–	-5.8			–	Gerling 2015
OU 3	Catacomb	Nevskoe	Kurgan 4, Burial 2			Indet.	Adult		0.7091		–		-5.5		–	Gerling 2015
OU 5	Catacomb	Nevskoe	Kurgan 4, Burial 3			Indet.	Adult			0.7092	–			-6.1	–	Gerling 2015
OU 4	Catacomb	Nevskoe	Kurgan 5, Burial 9			Male	18 years	0.7091			–	-4.0			–	Gerling 2015
**Average**								0.7101	0.7102	0.7099	0.0002	-4.9	-5.6	-5.5	0.8	

S1e Table. Isotope values for individuals from early sites of Kirovograd (KI), Peshtchanka (PE), Shakhta Stepnaya (SH), Vinogradnoe (VI), and Nevskoe (NE)

Period (site code) - compared to - Period (site code)	diff	p adj
Eneolithic (PE)-Eneolithic (VI)	-1.462	0.862022
Yamnaya (KI)-Eneolithic (VI)	-0.68	0.999968
Yamnaya (PE)-Eneolithic (VI)	-0.0725	1.000000
Yamnaya (SH)-Eneolithic (VI)	-0.4466667	1.000000
Yamnaya (VI)-Eneolithic (VI)	-0.2033333	1.000000
Early Catacomb (VI)-Eneolithic (VI)	-0.4557143	1.000000
Catacomb (KI)-Eneolithic (VI)	0.175	1.000000
Catacomb (PE)-Eneolithic (VI)	-0.095	1.000000
Catacomb (NE)-Eneolithic (VI)	-0.596	0.999998
Catacomb (SH)-Eneolithic (VI)	-0.0533333	1.000000
Catacomb (VI)-Eneolithic (VI)	-0.215	1.000000
Iron Age (AL)-Eneolithic (VI)	-0.89	0.999307
Iron Age (BM)-Eneolithic (VI)	-0.91	0.999924
Iron Age (DK)-Eneolithic (VI)	-0.46	1.000000
Iron Age (OR)-Eneolithic (VI)	-0.26	1.000000
Iron Age (ZB)-Eneolithic (VI)	-0.8266667	0.999649
Iron Age (ME)-Eneolithic (VI)	-1.11	0.972068
Iron Age (MG)-Eneolithic (VI)	-0.8715385	0.997103
Iron Age (BE)-Eneolithic (VI)	-1.77	0.308882
Yamnaya (KI)-Eneolithic (PE)	0.782	0.984634
Yamnaya (PE)-Eneolithic (PE)	1.3895	0.614600
Yamnaya (SH)-Eneolithic (PE)	1.0153333	0.982272
Yamnaya (VI)-Eneolithic (PE)	1.2586667	0.608151
Early Catacomb (VI)-Eneolithic (PE)	1.0062857	0.878124
Catacomb (KI)-Eneolithic (PE)	1.637	0.713215
Catacomb (PE)-Eneolithic (PE)	1.367	0.918438
Catacomb (NE)-Eneolithic (PE)	0.866	0.984926
Catacomb (SH)-Eneolithic (PE)	1.4086667	0.735679
Catacomb (VI)-Eneolithic (PE)	1.247	0.625199
Iron Age (AL)-Eneolithic (PE)	0.572	0.999931
Iron Age (BM)-Eneolithic (PE)	0.552	1.000000
Iron Age (DK)-Eneolithic (PE)	1.002	0.996790
Iron Age (OR)-Eneolithic (PE)	1.202	0.689494
Iron Age (ZB)-Eneolithic (PE)	0.6353333	0.999425
Iron Age (ME)-Eneolithic (PE)	0.352	1.000000
Iron Age (MG)-Eneolithic (PE)	0.5904615	0.996389
Iron Age (BE)-Eneolithic (PE)	-0.308	1.000000
Yamnaya (PE)-Yamnaya (KI)	0.6075	0.999736
Yamnaya (SH)-Yamnaya (KI)	0.2333333	1.000000
Yamnaya (VI)-Yamnaya (KI)	0.4766667	0.999952
Early Catacomb (VI)-Yamnaya (KI)	0.2242857	1.000000
Catacomb (KI)-Yamnaya (KI)	0.855	0.999134
Catacomb (PE)-Yamnaya (KI)	0.585	0.999997
Catacomb (NE)-Yamnaya (KI)	0.084	1.000000
Catacomb (SH)-Yamnaya (KI)	0.6266667	0.999903
Catacomb (VI)-Yamnaya (KI)	0.465	0.999967
Iron Age (AL)-Yamnaya (KI)	-0.21	1.000000
Iron Age (BM)-Yamnaya (KI)	-0.23	1.000000
Iron Age (DK)-Yamnaya (KI)	0.22	1.000000
Iron Age (OR)-Yamnaya (KI)	0.42	0.999993
Iron Age (ZB)-Yamnaya (KI)	-0.1466667	1.000000
Iron Age (ME)-Yamnaya (KI)	-0.43	0.999874
Iron Age (MG)-Yamnaya (KI)	-0.1915385	1.000000
Iron Age (BE)-Yamnaya (KI)	-1.09	0.120345
Yamnaya (SH)-Yamnaya (PE)	-0.3741667	1.000000
Yamnaya (VI)-Yamnaya (PE)	-0.1308333	1.000000
Early Catacomb (VI)-Yamnaya (PE)	-0.3832143	1.000000
Catacomb (KI)-Yamnaya (PE)	0.2475	1.000000
Catacomb (PE)-Yamnaya (PE)	-0.0225	1.000000
Catacomb (NE)-Yamnaya (PE)	-0.5235	0.999993
Catacomb (SH)-Yamnaya (PE)	0.0191667	1.000000
Catacomb (VI)-Yamnaya (PE)	-0.1425	1.000000
Iron Age (AL)-Yamnaya (PE)	-0.8175	0.996028
Iron Age (BM)-Yamnaya (PE)	-0.8375	0.999816
Iron Age (DK)-Yamnaya (PE)	-0.3875	1.000000
Iron Age (OR)-Yamnaya (PE)	-0.1875	1.000000
Iron Age (ZB)-Yamnaya (PE)	-0.7541667	0.997641
Iron Age (ME)-Yamnaya (PE)	-1.0375	0.831631
Iron Age (MG)-Yamnaya (PE)	-0.7990385	0.964870
Iron Age (BE)-Yamnaya (PE)	-1.6975	0.026637
Yamnaya (VI)-Yamnaya (SH)	0.2433333	1.000000
Early Catacomb (VI)-Yamnaya (SH)	-0.0090476	1.000000
Catacomb (KI)-Yamnaya (SH)	0.6216667	0.999999
Catacomb (PE)-Yamnaya (SH)	0.3516667	1.000000
Catacomb (NE)-Yamnaya (SH)	-0.1493333	1.000000
Catacomb (SH)-Yamnaya (SH)	0.3933333	1.000000
Catacomb (VI)-Yamnaya (SH)	0.2316667	1.000000
Iron Age (AL)-Yamnaya (SH)	-0.4433333	1.000000
Iron Age (BM)-Yamnaya (SH)	-0.4633333	1.000000
Iron Age (DK)-Yamnaya (SH)	-0.0133333	1.000000
Iron Age (OR)-Yamnaya (SH)	0.1866667	1.000000
Iron Age (ZB)-Yamnaya (SH)	-0.38	1.000000
Iron Age (ME)-Yamnaya (SH)	-0.6633333	0.999568
Iron Age (MG)-Yamnaya (SH)	-0.4248718	0.999999
Iron Age (BE)-Yamnaya (SH)	-1.3233333	0.500267

Early Catacomb (VI)-Yamnaya (VI)	-0.252381	1.000000	
Catacomb (KI)-Yamnaya (VI)	0.3783333	1.000000	
Catacomb (PE)-Yamnaya (VI)	0.1083333	1.000000	
Catacomb (NE)-Yamnaya (VI)	-0.3926667	1.000000	
Catacomb (SH)-Yamnaya (VI)	0.15	1.000000	
Catacomb (VI)-Yamnaya (VI)	-0.0116667	1.000000	
Iron Age (AL)-Yamnaya (VI)	-0.6866667	0.998387	
Iron Age (BM)-Yamnaya (VI)	-0.7066667	0.999964	
Iron Age (DK)-Yamnaya (VI)	-0.2566667	1.000000	
Iron Age (OR)-Yamnaya (VI)	-0.0566667	1.000000	
Iron Age (ZB)-Yamnaya (VI)	-0.6233333	0.999154	
Iron Age (ME)-Yamnaya (VI)	-0.9066667	0.820890	
Iron Age (MG)-Yamnaya (VI)	-0.6682051	0.967603	
Iron Age (BE)-Yamnaya (VI)	-1.5666667	0.006978	*
Catacomb (KI)-Early Catacomb (VI)	0.6307143	0.999992	
Catacomb (PE)-Early Catacomb (VI)	0.3607143	1.000000	
Catacomb (NE)-Early Catacomb (VI)	-0.1402857	1.000000	
Catacomb (SH)-Early Catacomb (VI)	0.402381	1.000000	
Catacomb (VI)-Early Catacomb (VI)	0.2407143	1.000000	
Iron Age (AL)-Early Catacomb (VI)	-0.4342857	0.999997	
Iron Age (BM)-Early Catacomb (VI)	-0.4542857	1.000000	
Iron Age (DK)-Early Catacomb (VI)	-0.0042857	1.000000	
Iron Age (OR)-Early Catacomb (VI)	0.1957143	1.000000	
Iron Age (ZB)-Early Catacomb (VI)	-0.3709524	0.999999	
Iron Age (ME)-Early Catacomb (VI)	-0.6542857	0.984148	
Iron Age (MG)-Early Catacomb (VI)	-0.4158242	0.999789	
Iron Age (BE)-Early Catacomb (VI)	-1.3142857	0.031229	
Catacomb (PE)-Catacomb (KI)	-0.27	1.000000	
Catacomb (NE)-Catacomb (KI)	-0.771	0.999909	
Catacomb (SH)-Catacomb (KI)	-0.2283333	1.000000	
Catacomb (VI)-Catacomb (KI)	-0.39	1.000000	
Iron Age (AL)-Catacomb (KI)	-1.065	0.993336	
Iron Age (BM)-Catacomb (KI)	-1.085	0.999096	
Iron Age (DK)-Catacomb (KI)	-0.635	1.000000	
Iron Age (OR)-Catacomb (KI)	-0.435	1.000000	
Iron Age (ZB)-Catacomb (KI)	-1.0016667	0.995696	
Iron Age (ME)-Catacomb (KI)	-1.285	0.896574	
Iron Age (MG)-Catacomb (KI)	-1.0465385	0.976904	
Iron Age (BE)-Catacomb (KI)	-1.945	0.164999	
Catacomb (NE)-Catacomb (PE)	-0.501	1.000000	
Catacomb (SH)-Catacomb (PE)	0.0416667	1.000000	
Catacomb (VI)-Catacomb (PE)	-0.12	1.000000	
Iron Age (AL)-Catacomb (PE)	-0.795	0.999858	
Iron Age (BM)-Catacomb (PE)	-0.815	0.999986	
Iron Age (DK)-Catacomb (PE)	-0.365	1.000000	
Iron Age (OR)-Catacomb (PE)	-0.165	1.000000	
Iron Age (ZB)-Catacomb (PE)	-0.7316667	0.999940	
Iron Age (ME)-Catacomb (PE)	-1.015	0.989125	
Iron Age (MG)-Catacomb (PE)	-0.7765385	0.999354	
Iron Age (BE)-Catacomb (PE)	-1.675	0.410690	
Catacomb (SH)-Catacomb (NE)	0.5426667	0.999997	
Catacomb (VI)-Catacomb (NE)	0.381	1.000000	
Iron Age (AL)-Catacomb (NE)	-0.294	1.000000	
Iron Age (BM)-Catacomb (NE)	-0.314	1.000000	
Iron Age (DK)-Catacomb (NE)	0.136	1.000000	
Iron Age (OR)-Catacomb (NE)	0.336	1.000000	
Iron Age (ZB)-Catacomb (NE)	-0.2306667	1.000000	
Iron Age (ME)-Catacomb (NE)	-0.514	0.999820	
Iron Age (MG)-Catacomb (NE)	-0.2755385	1.000000	
Iron Age (BE)-Catacomb (NE)	-1.174	0.293929	
Catacomb (VI)-Catacomb (SH)	-0.1616667	1.000000	
Iron Age (AL)-Catacomb (SH)	-0.8366667	0.998161	
Iron Age (BM)-Catacomb (SH)	-0.8566667	0.999881	
Iron Age (DK)-Catacomb (SH)	-0.4066667	1.000000	
Iron Age (OR)-Catacomb (SH)	-0.2066667	1.000000	
Iron Age (ZB)-Catacomb (SH)	-0.7733333	0.998995	
Iron Age (ME)-Catacomb (SH)	-1.0566667	0.917142	
Iron Age (MG)-Catacomb (SH)	-0.8182051	0.987898	
Iron Age (BE)-Catacomb (SH)	-1.7166667	0.095908	
Iron Age (AL)-Catacomb (VI)	-0.675	0.998709	
Iron Age (BM)-Catacomb (VI)	-0.695	0.999972	
Iron Age (DK)-Catacomb (VI)	-0.245	1.000000	
Iron Age (OR)-Catacomb (VI)	-0.045	1.000000	
Iron Age (ZB)-Catacomb (VI)	-0.6116667	0.999344	
Iron Age (ME)-Catacomb (VI)	-0.895	0.836128	
Iron Age (MG)-Catacomb (VI)	-0.6565385	0.972776	
Iron Age (BE)-Catacomb (VI)	-1.555	0.007811	*
Iron Age (BM)-Iron Age (AL)	-0.02	1.000000	
Iron Age (DK)-Iron Age (AL)	0.43	1.000000	
Iron Age (OR)-Iron Age (AL)	0.63	0.999488	
Iron Age (ZB)-Iron Age (AL)	0.0633333	1.000000	
Iron Age (ME)-Iron Age (AL)	-0.22	1.000000	
Iron Age (MG)-Iron Age (AL)	0.0184615	1.000000	
Iron Age (BE)-Iron Age (AL)	-0.88	0.802151	

Iron Age (DK)-Iron Age (BM)	0.45	1.000000
Iron Age (OR)-Iron Age (BM)	0.65	0.999990
Iron Age (ZB)-Iron Age (BM)	0.0833333	1.000000
Iron Age (ME)-Iron Age (BM)	-0.2	1.000000
Iron Age (MG)-Iron Age (BM)	0.0384615	1.000000
Iron Age (BE)-Iron Age (BM)	-0.86	0.997246
Iron Age (OR)-Iron Age (DK)	0.2	1.000000
Iron Age (ZB)-Iron Age (DK)	-0.3666667	1.000000
Iron Age (ME)-Iron Age (DK)	-0.65	0.999972
Iron Age (MG)-Iron Age (DK)	-0.4115385	1.000000
Iron Age (BE)-Iron Age (DK)	-1.31	0.828477

Iron Age (ZB)-Iron Age (OR)	-0.5666667	0.999773	
Iron Age (ME)-Iron Age (OR)	-0.85	0.887845	
Iron Age (MG)-Iron Age (OR)	-0.6115385	0.987045	
Iron Age (BE)-Iron Age (OR)	-1.51	0.011966	*
Iron Age (ME)-Iron Age (ZB)	-0.2833333	1.000000	
Iron Age (MG)-Iron Age (ZB)	-0.0448718	1.000000	
Iron Age (BE)-Iron Age (ZB)	-0.9433333	0.557939	
Iron Age (MG)-Iron Age (ME)	0.2384615	0.999999	
Iron Age (BE)-Iron Age (ME)	-0.66	0.699633	
Iron Age (BE)-Iron Age (MG)	-0.8984615	0.009608	*

	Df	Sum Sq	Mean Sq	F value	Pr(>F)
Site	19	50.15	2.6395	3.729	4.04E-06
Residuals	127	89.90	0.7079		

S2a Table. Average oxygen isotope values of individuals compared between sites (separated by time period) using ANOVA and TukeyHSD (95% confidence) and a summary of this comparison of sites (separated by time period)

Period	diff	p adj	
Yamnaya-Eneolithic	0.6495238	0.477090	
Early Catacomb-Eneolithic	0.5885714	0.745367	
Catacomb-Eneolithic	0.8070635	0.275370	
Iron Age-Eneolithic	-0.1433739	0.994430	
Early Catacomb-Yamnaya	-0.0609524	0.999874	
Catacomb-Yamnaya	0.1575397	0.983087	
Iron Age-Yamnaya	-0.7928977	0.003811	*
Catacomb-Early Catacomb	0.2184921	0.983080	
Iron Age-Early Catacomb	-0.7319453	0.246249	
Iron Age-Catacomb	-0.9504374	0.000758	*

	Df	Sum Sq	Mean Sq	F value	Pr(>F)
Period	4	22.53	5.634	6.807	4.84E-05
Residuals	142	117.52	0.828		

S2b Table. Average oxygen isotope values of individuals compared between periods using ANOVA and TukeyHSD (95% confidence) and a summary of this comparison of periods

Site - compared to - Site	diff	p adj
Mamai Gora-Medvin	-2.9339744	0.000447
Alexandropol-Medvin	-4.4616667	0.000470
Babina Mogila-Medvin	-6.0416667	0.001197
Drana Khota-Medvin	-5.2416667	0.008247
Ordzhonikidze-Medvin	-3.5416667	0.005596
Zolotaya Balka-Medvin	-3.9083333	0.001435
Belsk-Medvin	-1.9122549	0.053059
Alexandropol-Mamai Gora	-1.5276923	0.690448
Babina Mogila-Mamai Gora	-3.1076923	0.308833
Drana Khota-Mamai Gora	-2.3076923	0.684763
Ordzhonikidze-Mamai Gora	-0.6076923	0.995938
Zolotaya Balka-Mamai Gora	-0.974359	0.939396
Belsk-Mamai Gora	1.0217195	0.407883
Babina Mogila-Alexandropol	-1.58	0.969643
Drana Khota-Alexandropol	-0.78	0.999609
Ordzhonikidze-Alexandropol	0.92	0.991326
Zolotaya Balka-Alexandropol	0.5533333	0.999657
Belsk-Alexandropol	2.5494118	0.088858
Drana Khota-Babina Mogila	0.8	0.999859
Ordzhonikidze-Babina Mogila	2.5	0.713174
Zolotaya Balka-Babina Mogila	2.1333333	0.847629
Belsk-Babina Mogila	4.1294118	0.054583
Ordzhonikidze-Drana Khota	1.7	0.948803
Zolotaya Balka-Drana Khota	1.3333333	0.986702
Belsk-Drana Khota	3.3294118	0.218649
Zolotaya Balka-Ordzhonikidze	-0.3666667	0.999970
Belsk-Ordzhonikidze	1.6294118	0.492261
Belsk-Zolotaya Balka	1.9960784	0.235071

	Df	Sum Sq	Mean Sq	F value	Pr(>F)
Site	7	169.4	24.198	7.103	1.13E-06
Residuals	85	289.6	3.407		

S2c Table. Average carbon isotope values of individuals compared between Iron Age sites using ANOVA and TukeyHSD (95% confidence) and a summary of this comparison of periods

Site	Period(s)	Average Sr ratio	Median Sr ratio	3MAD$_{norm}$	Baseline range (3MAD$_{norm}$) from	Baseline range (3MAD$_{norm}$) to	# outlying values / # outlying individuals	% outlying individuals	Total number of individuals analyzed	Located in which substrate	Local Sr ratios	Difference between Sr ratio and human *average* Sr	Materials measured for Sr ratios	Reference
Vinogradnoe	Eneolithic, Yamnaya, Early Catacomb, Catacomb	0.7098	0.7098	0.0003	0.70951	0.71013	3 / 3	18.8	16	Cenozoic	0.7076	0.0022	rodent tooth (modern)	Gerling 2015
											0.7119	0.0021	shell (modern)	Gerling 2015
											0.7101	0.0003	rodent tooth (modern)	Gerling 2015
											0.7097	0.0001	medieval dog tooth	Gerling 2015
											0.7097	0.0001	medieval dog tooth	Gerling 2015
											0.7103	0.0005	plant (modern)	Gerling 2015
Peshtchanka	Eneolithic, Yamnaya, Catacomb	0.7103	0.7104	0.0008	0.70957	0.71118	1 / 1	14.3	7	Cenozoic	0.7114	0.0011	cattle 18th century	Gerling 2015
											0.7116	0.0013	cattle 18th century	Gerling 2015
											0.7102	0.0001	plant (modern)	Gerling 2015
Kirovograd	Yamnaya, Catacomb	0.7107	0.7107	0.0016	0.70912	0.71232	0 / 0	0.0	8	Cenozoic	0.7104	0.0003	horse tooth (ancient)	Gerling 2015
											0.7104	0.0003	horse tooth (ancient)	Gerling 2015
											0.7103	0.0004	horse tooth (ancient)	Gerling 2015
											0.7104	0.0003	horse tooth (ancient)	Gerling 2015
											0.7103	0.0004	horse tooth (ancient)	Gerling 2015
											0.7102	0.0005	snail (modern)	Gerling 2015
											0.7101	0.0006	oak tree leaves	Gerling 2015
Shakhta Stepnaya	Yamnaya, Catacomb	0.7099	0.7100	0.0001	0.70987	0.71005	1 / 1	25.0	4	Cenozoic	0.7091	0.0008	shell (ancient)	Gerling 2015
											0.7109	0.0010	oak tree leaves	Gerling 2015
Nevskoe	Catacomb	0.7091	0.7091	0.0004	0.70876	0.70948	0 / 0	0.0	5	Mesozoic				Gerling 2015
Alexandropol	Iron Age/Scythian	0.7099	0.7099	0.0006	0.70936	0.71047	0 / 0	0.0	5	Cenozoic	0.7102	0.0003	rodent tooth (modern)	Gerling 2015
Babina Mogila	Iron Age/Scythian	0.7097	0.7097	0.0010	0.70876	0.71068	0 / 0	0.0	2	Cenozoic				Gerling 2015
Drana Kokhta	Iron Age/Scythian	0.7103	0.7103	0.0020	0.70834	0.71224	0 / 0	0.0	2	Cenozoic				Gerling 2015
Ordzhonikidze	Iron Age/Scythian	0.7100	0.7100	0.0003	0.70964	0.71026	0 / 0	0.0	6	Cenozoic	0.7098	0.0002	sheep bone (ancient)	Gerling 2015
Zolotaya Balka	Iron Age/Scythian	0.7098	0.7099	0.0005	0.70945	0.71036	1 / 1	16.7	6	Cenozoic				Gerling 2015
Medvin	Iron Age/Scythian	0.7108	0.7108	0.0003	0.7104	0.7111	0 / 0	0.0	9	Cenozoic				This study
Mamai-Gora	Iron Age/Scythian	0.7100	0.7099	0.0003	0.7096	0.7103	4 / 2	15.4	13	Cenozoic				This study
Bel'sk	Iron Age/Scythian	0.7099	0.7102	0.0010	0.70920	0.71120	6 / 5	25.0	20	Cenozoic	0.7102	0.0002	rodent tooth	Ventresca Miller et al. 2019

S3 Table. Average strontium isotope ratios for all sites, with baseline values and outliers identified

Site	r	r square	df	sample size	p-value	
Medvin	0.45	0.20	10	12	0.1407	
Mamai-Gora	0.33	0.11	23	25	0.1083	
Bel'sk	0.41	0.17	32	34	0.0153	*
Alexandropol	0.31	0.09	3	5	0.6143	
Babina Mogila	-	-	-	2	-	
Drana Kokhta	-	-	-	2	-	
Ordzhonikidze	0.28	0.08	4	6	0.5948	
Zolotaya Balka	0.47	0.22	4	6	0.3482	

S4 Table. Linear Regression analysis of the relationship between strontium and carbon isotope values for Scythian era sites

Site	Sample Number	Laboratory Designation	Uncalibrated dates	Calibrated	Notes
Mamai-Gora	Mamai-Gora 183	Poz-93203	**2265 ± 30 BP**	**399-209 cal BC**	2,7%N 8,4%C, 11.3%coll
Mamai-Gora	Mamai-Gora 212	Poz-93205	**2195 ± 35 BP**	**369-174 cal BC**	3,4%N 10,1%C, 4.7%coll
Medvin	Medvin 233	Poz-93201	**2480 ± 35 BP**	**775-431 cal BC**	1%N 6,5%C, 5%coll
Medvin	Medvin 230	Poz-93202	**2320 ± 35 BP**	**484-233 cal BC**	0,7%N 5,5%C, 5.4%coll

S5 Table. Radiocarbon dates of human remains from Mamai-Gora and Medvin

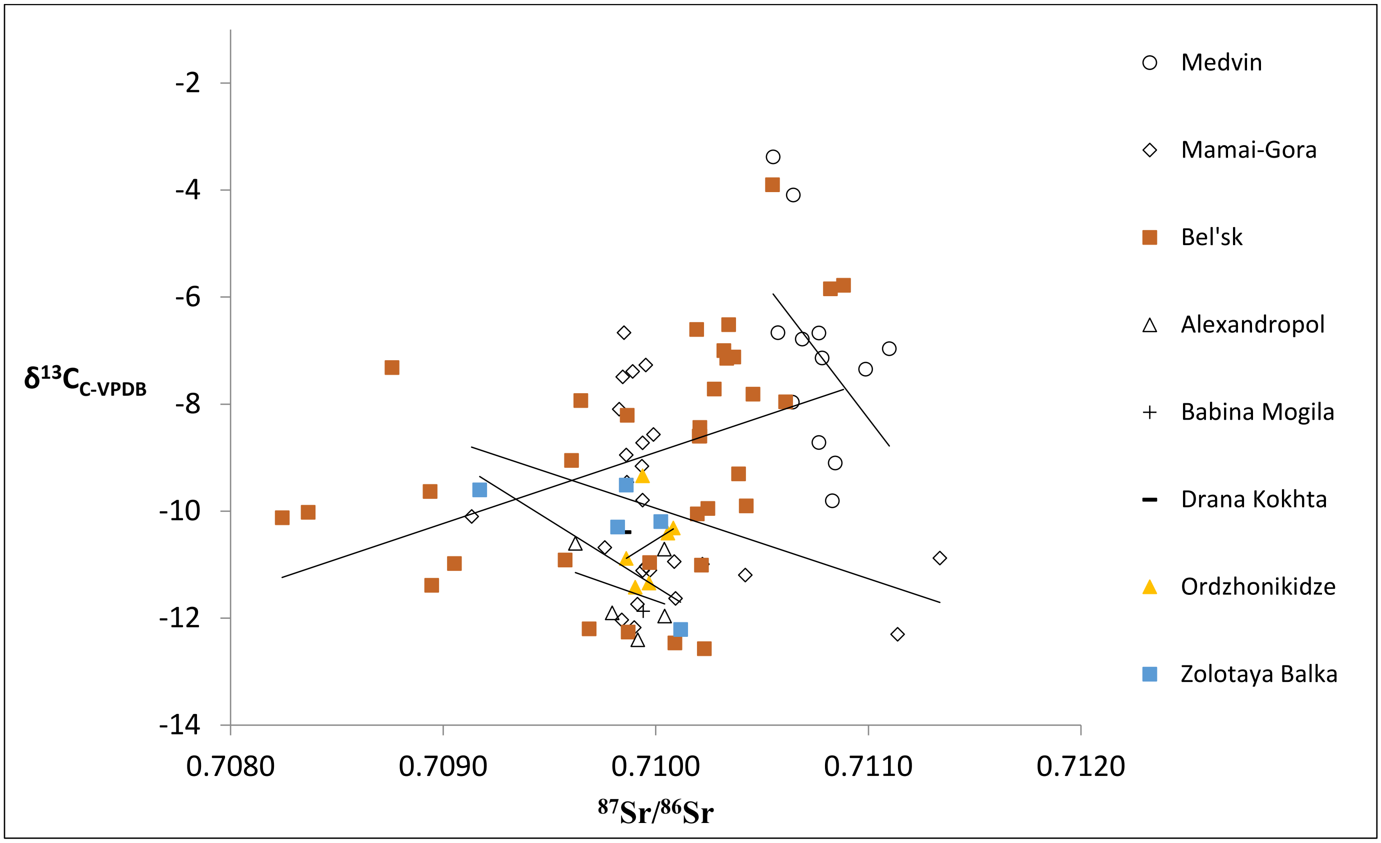

S1 Fig. Correlation of carbon and strontium isotope ratios at Scythian era sites
